# Imatinib blocks tyrosine phosphorylation of Smad4 and restores TGF-β growth-suppressive signaling in BCR-ABL1-positive leukemia

**DOI:** 10.1038/s41392-023-01327-5

**Published:** 2023-03-24

**Authors:** Lijing Wang, Shuchen Gu, Fenfang Chen, Yi Yu, Jin Cao, Xinran Li, Chun Gao, Yanzhen Chen, Shuchong Yuan, Xia Liu, Jun Qin, Bin Zhao, Pinglong Xu, Tingbo Liang, Hongyan Tong, Xia Lin, Xin-Hua Feng

**Affiliations:** 1 The MOE Key Laboratory of Biosystems Homeostasis & Protection and Zhejiang Provincial Key Laboratory of Cancer Molecular Cell Biology, Life Sciences Institute, Zhejiang University, Hangzhou, Zhejiang 310058 China; 2 Center for Life Sciences, Shaoxing Institute, Zhejiang University, Shaoxing, Zhejiang 321000 China; 3 Cancer Center, Zhejiang University, Hangzhou, Zhejiang 310058 China; 4 ZJU-Hangzhou Global Scientific and Technological Innovation Center, Zhejiang University, Hangzhou, Zhejiang 311200 China; 5 Department of Hematology, The First Affiliated Hospital, Zhejiang University School of Medicine, Hangzhou, 310003 China; 6 Beijing Proteome Research Center, National Center for Protein Sciences, Beijing, China; 7 Department of Hepatobiliary and Pancreatic Surgery and Zhejiang Provincial Key Laboratory of Pancreatic Disease, The First Affiliated Hospital, Zhejiang University School of Medicine, Hangzhou, 310003 China; 8 The Second Affiliated Hospital, Zhejiang University, Hangzhou, Zhejiang 310009 China

**Keywords:** Haematological cancer, Cell biology

## Abstract

Loss of TGF-β-mediated growth suppression is a major contributor to the development of cancers, best exemplified by loss-of-function mutations in genes encoding components of the TGF-β signaling pathway in colorectal and pancreatic cancers. Alternatively, gain-of-function oncogene mutations can also disrupt antiproliferative TGF-β signaling. However, the molecular mechanisms underlying oncogene-induced modulation of TGF-β signaling have not been extensively investigated. Here, we show that the oncogenic BCR-ABL1 of chronic myelogenous leukemia (CML) and the cellular ABL1 tyrosine kinases phosphorylate and inactivate Smad4 to block antiproliferative TGF-β signaling. Mechanistically, phosphorylation of Smad4 at Tyr195, Tyr301, and Tyr322 in the linker region interferes with its binding to the transcription co-activator p300/CBP, thereby blocking the ability of Smad4 to activate the expression of cyclin-dependent kinase (CDK) inhibitors and induce cell cycle arrest. In contrast, the inhibition of BCR-ABL1 kinase with Imatinib prevented Smad4 tyrosine phosphorylation and re-sensitized CML cells to TGF-β-induced antiproliferative and pro-apoptotic responses. Furthermore, expression of phosphorylation-site-mutated Y195F/Y301F/Y322F mutant of Smad4 in *Smad4*-null CML cells enhanced antiproliferative responses to TGF-β, whereas the phosphorylation-mimicking Y195E/Y301E/Y322E mutant interfered with TGF-β signaling and enhanced the in vivo growth of CML cells. These findings demonstrate the direct role of BCR-ABL1 tyrosine kinase in suppressing TGF-β signaling in CML and explain how Imatinib-targeted therapy restored beneficial TGF-β anti-growth responses.

## Introduction

TGF-β and related cytokines mediate a plethora of cellular processes including proliferation, differentiation, apoptosis, and immune surveillance.^1–3^ Upon ligand engagement, the type II receptor (TβRII) phosphorylates the cytoplasmic domain of the type I receptor (TβRI) to recruit and phosphorylate the downstream Smad2 and Smad3. After phosphorylation, Smad2/3 forms a trimeric complex with Smad4 and transduces its transcriptional functions.^1,4^ This process is sophisticatedly modulated in a context-dependent manner.^5^ The Smad complex interacts with various transcription factors such as Runx3^6^ and FOXL2^7^ and/or co-activators such as p300^8^ and BRD7^9^ to regulate the transcription of TGF-β-responsive genes (e.g., p21^CIP1^ ^10^, p15^INK4b^ ^11^, and p57^KIP2^ ^12^) in various cell types. Termination of Smad-dependent signaling is achieved through the dephosphorylation and nuclear export of R-Smads.^13,14^

Dysregulation of TGF-β signaling is often observed during cancer initiation and progression.^1–3^ Genetic inactivation of TβRII occurs in approximately 25% of colorectal cancer.^15^ Smad4, the central component of TGF-β signaling, has frequently been deleted in sporadic colorectal cancer and pancreatic cancer.^16^ However, the TGF-β pathway remains relatively intact in most cancer types, possibly because cancer cells benefit from signaling for subsequent malignancy. In some cases of leukemia, resistance to TGF-β-induced cytostatic effects was correlated with the activation of oncogenic transcription factors,^17^ for example, Runx1-ETO in acute myeloid leukemia (AML)^18^ and PML-RARα in acute promyelocytic leukemia (APL) and Evi-1 in chronic myeloid leukemia (CML).^3^ Amplified Evi-1 in CML interacts with Smad3 and disrupts Smad3-DNA binding, leading to repression of TGF-β signaling in the blast phase of CML.^19^ Therefore, oncogene-mediated blockade of TGF-β signaling might be an alternative way for cancer cells to escape TGF-β tumor suppressor signaling.

BCR-ABL1 fusion gene is a hallmark of CML. Malignancy induced by the BCR-ABL1 fusion gene accounts for more than 90% of the incidence of CML.^20^ In addition to CML, approximately 25–30% of adult B-cell ALL cases are BCR-ABL1-positive,^21^ and 2–10% of children’s ALL cases.^20^ The ubiquitously expressed non-receptor tyrosine kinase ABL1 plays a vital role in controlling cell proliferation and survival. Under normal circumstances, its kinase activity is auto-regulated and maintained at an average level to prevent oncogenic events in leukemia.^21^ However, constitutively activated BCR-ABL1, a gene fusion product resulting from chromosomal translocation,^20^ leads to uncontrolled growth and survival of hematopoietic stem cells and initiates CML pathogenesis. BCR-ABL1 tyrosine kinase inhibitors (TKIs) are broadly applied in the treatment of BCR-ABL1-positive CML, particularly in the chronic phase.^22^ Five TKIs (imatinib, dasatinib, nilotinib, bosutinib, ponatinib, and radotinib) are currently worldwide approved, among which imatinib (Gleevec or STI571) is the first generation TKI and a strong competitive inhibitor of ATP binding to ABL1 kinase. However, in advanced or blast phases, TKIs are less efficient, even when they are administered in higher doses.^23^ Besides, resistance to Imatinib and other BCR-ABL1 inhibitors remains a clinical challenge, especially in patients with advanced disease. Therefore, exploring the underlying mechanisms of malignancy and resistance related to BCR-ABL1 may provide insights into predicting long-term responses and new drug development.

In the current study, we aimed to explore the molecular events that evade the tumor-suppressive function of TGF-β and contribute to CML pathogenesis. Given that tyrosine kinases are often constitutively active in various cancers and drive oncogenesis and may act to inhibit TGF-β tumor-suppressive signaling, we screened tyrosine kinases for tyrosine phosphorylation of the key TGF-β signal transducer Smad4. Intriguingly, we discovered that ABL1 can phosphorylate Smad4 and interfere with its interaction with p300/CBP, thereby impairing the ability of Smad4 to mediate TGF-β-induced cell cycle arrest. Conversely, the ABL1 inhibitor Imatinib prevented Smad4 tyrosine phosphorylation and enabled TGF-β-insensitive, BCR-ABL1-positive K562 cells to achieve restored or enhanced TGF-β responses. Accordingly, our study not only determined a new consequence of BCR-ABL1 but also revealed a novel mechanism of Imatinib in treating CML.

## Results

### ABL kinases attenuate TGF-β-induced responses

**Fig. 1:**
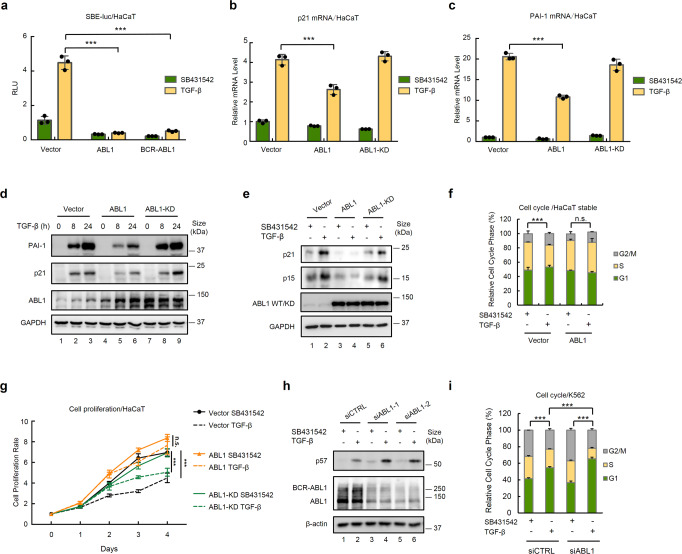
ABL kinases attenuate TGF-β-induced responses. **a** ABL kinases abolish TGF-β-induced SBE-luc reporter gene activity. HaCaT cells were transfected with expression plasmids for SBE-luc, Renilla-luc (internal control), and ABL1 or BCR-ABL1 as indicated and treated with 2 ng/ml of TGF-β or 5 μM SB431542 (TGF-β inhibitor) for 8 h. Relative luciferase activity was measured as described in the Materials and Methods. Data are shown as mean ± SEM; *n* = 3. Statistical analysis was performed using ANOVA. ***P < 0.001. **b**–**d** ABL1 inhibits TGF-β-induced expression of p21 and PAI-1. HaCaT cells stably expressing ABL1 or ABL1-KD were treated with TGF-β (2 ng/ml) for 12 h (for RNA analysis) and 24 h (for protein analysis). Total RNAs were extracted and subjected to qRT-PCR analysis using primers specific to p21 (**b**) and PAI-1 (**c**). Data are shown as mean ± SEM; *n* = 3. Statistical analysis was performed using ANOVA. ***P < 0.001. **d** Cell lysates were prepared and subjected to western blotting analysis with appropriate antibodies as indicated. **e** ABL1 inhibits TGF-β-induced p21 and p15 expression. Jurkat cells were transfected with expression plasmids for ABL1 or ABL1-KD and treated with TGF-β (2 ng/ml) for 24 h. Cell lysate preparation and western blotting analysis were carried out as described in **d**. **f** ABL1 attenuates TGF-β-induced cell cycle arrest. HaCaT cells stably expressing ABL1 were treated with TGF-β (2 ng/ml) or SB431542 (5 μM) for 48 h. The percentage of cell populations in each cell cycle phase was determined by FACS. Statistical analysis showing cells in G1 stage was performed using ANOVA. ***P < 0.001. **g** ABL1 attenuates TGF-β-induced growth inhibitory effect. HaCaT cells stably expressing ABL1 or ABL1-KD were treated with TGF-β (2 ng/ml) or SB431542 (5 μM) for indicated days. Cell proliferation was determined by using CCK8 assay. On *Y* axis, cell proliferation rate indicates the fold change from Day 0. Data are shown as mean ± SEM; *n* = 3. Statistical analysis was performed using ANOVA. ***P < 0.001. n.s. represents no significance. **h** Knockdown of ABL1 promotes TGF-β-induced p57 expression. K562 cells were transfected with two independent siRNAs against ABL1 individually and treated with TGF-β (2 ng/ml) for 24 h. Cell lysate preparation and western blotting analysis were carried out as described in **d**. **i** Knockdown of ABL1 enhances TGF-β-induced cell cycle arrest in K562 cells. Cell transfection and treatment were done as described in **h**. The percentage of cell populations in each cell cycle phase was determined by FACS. Statistical analysis showing cells in G1 stage was performed using ANOVA. ***P < 0.001

TGF-β signaling potently suppresses cell proliferation and promotes apoptosis in early-stage cancerous cells, whereas protein tyrosine kinases (PTKs) often positively regulate cell growth and survival. To search for novel regulators that antagonize TGF-β signaling in cancer, we performed a cDNA screen on PTKs that influence TGF-β signaling. Among the 82 tyrosine kinases tested (data not shown), ABL1 was a strong candidate for inhibiting TGF-β-mediated transcriptional responses and phosphorylated Smad4 (Supplementary Fig. 1a and described below). Constitutively active BCR-ABL1 derived from CML had the same effect. Using the Smad-responsive SBE-Luc reporter assay,^24^ we found that ectopic expression of either ABL1 or BCR-ABL1 completely suppressed TGF-β-induced transcriptional activity in highly TGF-β-responsive human keratinocyte HaCaT cells (Fig. 1a). Consistently, the stable expression of ABL1 also decreased TGF-β-induced transcription of the CDK2 inhibitor p21 (Fig. 1b) and PAI-1 (an extracellular matrix protein) (Fig. 1c) by nearly 50% in HaCaT cells. Notably, ABL1-KD, a kinase-dead mutant of ABL1 (Supplementary Fig. 1b), failed to attenuate the TGF-β-induced upregulation of p21 and PAI-1 mRNAs (Fig. 1b, c) and proteins (Fig. 1d). Similarly, stable expression of wild-type BCR-ABL1, but not BCR-ABL1-KD, abrogated TGF-β-induced expression of CDK4/6 inhibitor p15 in HaCaT cells (Supplementary Fig. 1c) and Jurkat cells (Fig. 1e). Consistently, ectopic expression of ABL1 abrogated TGF-β-induced G1 arrest (Fig. 1f) and promoted the proliferation of HaCaT cells (Fig. 1g).

To further validate the regulatory activity of ABL1 on TGF-β signaling, endogenous BCR-ABL1 expression was knocked down using two specific siRNAs in BCR-ABL1-positive human leukemia K562 cells. BCR-ABL1 knockdown significantly enhanced TGF-β-induced SBE-Luc reporter response (Supplementary Fig. 1d). In agreement with the fact that TGF-β often induces the expression of the CDK2 inhibitor p57 in hematopoietic cells, siBCR-ABL1 increased TGF-β-induced p57 expression (Fig. 1h). In addition, although TGF-β only slightly changed the cell cycle profile in control K562 cells, more significant G1 arrest was observed in siABL1 cells (Fig. 1i). Taken together, these results demonstrate that BCR-ABL1 and ABL1 attenuate TGF-β signaling.

### Imatinib enhances TGF-β anti-growth responses

**Fig. 2:**
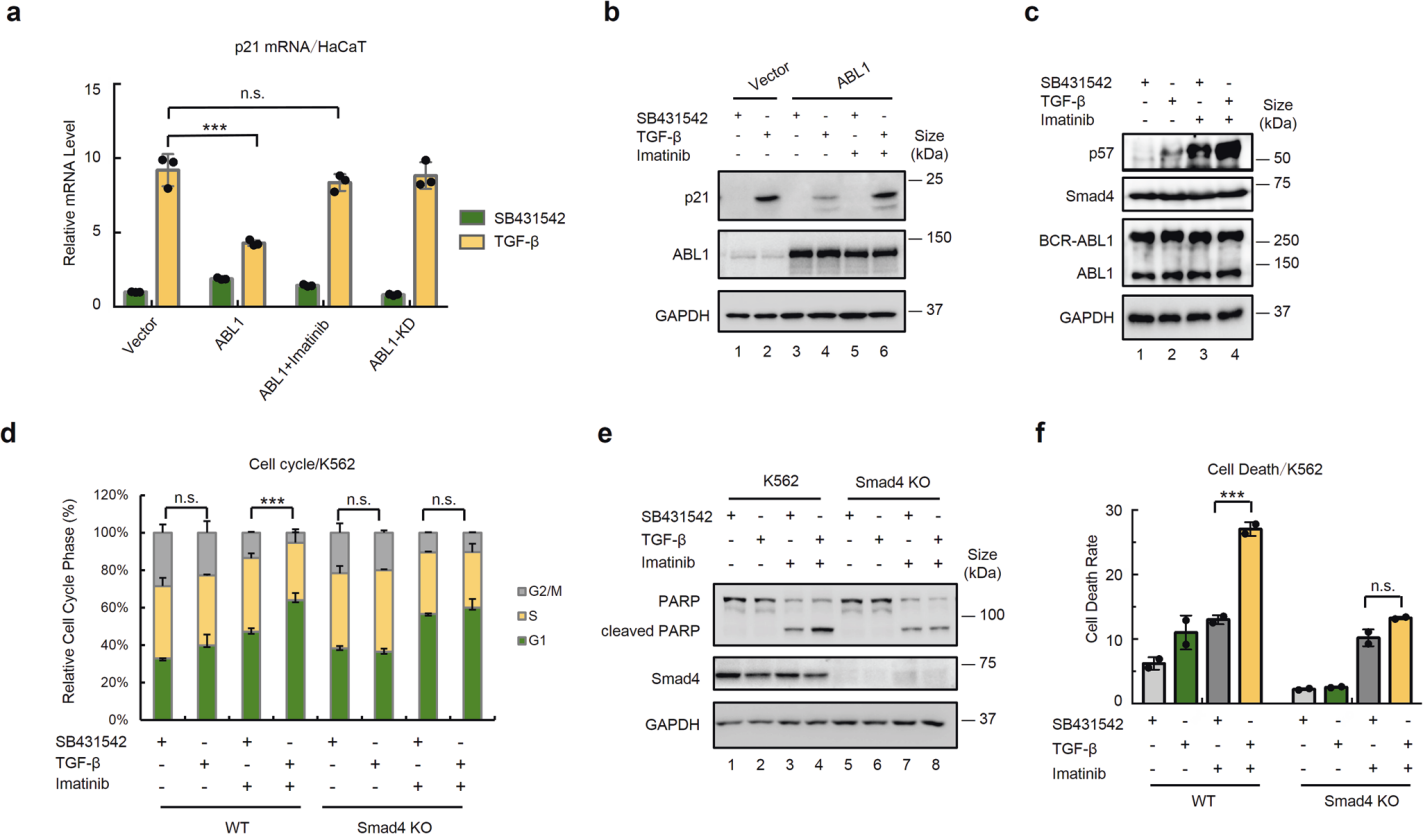
Imatinib restores ABL1-suppressed TGF-β responses. **a**, **b** Imatinib reverses ABL1-mediated suppression of TGF-β-induced p21 expression. HaCaT cells stably expressing ABL1 or ABL1-KD were treated with TGF-β (2 ng/ml), SB431542 (5 μM) and/or Imatinib (0.5 μM) for 12 h (for RNA analysis) and 24 h (for protein analysis). **a** Cells were harvested for RNA extraction and qRT-PCR analysis using primers specific to p21. Data are shown as mean ± SEM; *n* = 3. Statistical analysis was performed using ANOVA. ***P < 0.001. **b** Cell lysates were prepared and subjected to western blotting analysis with anti-p21 antibody and other appropriate antibodies as indicated. **c** Imatinib enhances TGF-β-induced p57 expression. K562 cells were similarly treated as described in **b**. Cell lysates were prepared and subjected to western blotting analysis with anti-p57 antibody and other appropriate antibodies as indicated. **d** Imatinib facilitates TGF-β-induced G1 arrest in a Smad4-dependent manner. Wild-type (WT) or Smad4^−/−^ K562 cells were similarly treated as described in **a**. FACS analysis was done as described in Fig. 1i. Statistical analysis showing cells in G1 stage was performed using ANOVA. ***P < 0.001. **e** TGF-β and Imatinib synergize to induce PARP cleavage dependent on Smad4. Cell treatment, lysate preparation, and western blotting analysis were carried out as described in **d**. **f** TGF-β and Imatinib synergize to induce cell death dependent on Smad4. WT or Smad4−/− K562 cells were similarly treated as described in **a**. The proportion of dead cells was measured by FACS and the sub G1 peak in the cell cycle distribution analysis indicated dead cells. Statistical analysis was performed using ANOVA. ***P < 0.001

Imatinib, a small molecule inhibitor targeting ABL kinase, has been widely used in the clinical treatment of CML and other Philadelphia-positive (Ph+) leukemia expressing BCR-ABL1.^25,26^ Since ABL kinases attenuate TGF-β signaling, we reasoned that Imatinib could restore or enhance TGF-β-induced responses in ABL1-positive cancer cells. Indeed, Imatinib reversed the antagonizing effect of ABL1 on TGF-β-induced p21 expression in HaCaT cells (Fig. 2a, b). Yet, Imatinib had no effects on TGF-β responses in parental HaCaT cells (Supplementary Fig. 2a). Similarly, Imatinib augmented TGF-β-induced expression of p57 (Fig. 2c) and markedly promoted TGF-β-induced G1 arrest in K562 cells (Fig. 2d, Supplementary Fig. 2b), similar to the effect observed with ABL1 knockdown. This enhanced TGF-β-induced G1 arrest was Smad4-dependent, as the TGF-β-induced effects disappeared in Smad4−/− cells (Fig. 2d). It has been reported that Imatinib induces apoptosis of BCR-ABL1-expressing cells such as K562,^27,28^ while TGF-β can also exert pro-apoptotic effects by inducing apoptosis in certain cell types. Hence, we wondered whether Imatinib can sensitize cells to a higher TGF-β apoptotic response. Indeed, Imatinib treatment enabled TGF-β to induce more apoptosis, as shown by the increased cleavage of PARP in K562 cells (Fig. 2e). However, TGF-β had no additional effect on Imatinib-induced G1 arrest and PARP cleavage in Smad4−/− cells (Fig. 2d, e), indicating that Smad4 is required for these processes. Furthermore, FACS analysis showed that Imatinib sensitized K562 cells to achieve a higher TGF-β-induced death rate in WT (wildtype) K562 cells but not in Smad4−/− K562 cells (Fig. 2f). Together, these results indicate that ABL inhibition sensitizes BCR-ABL1-positive tumor cells to TGF-β-induced growth inhibition and apoptosis.

### ABL kinases phosphorylate Smad4 on Tyr195, Tyr301, and Tyr322

**Fig. 3:**
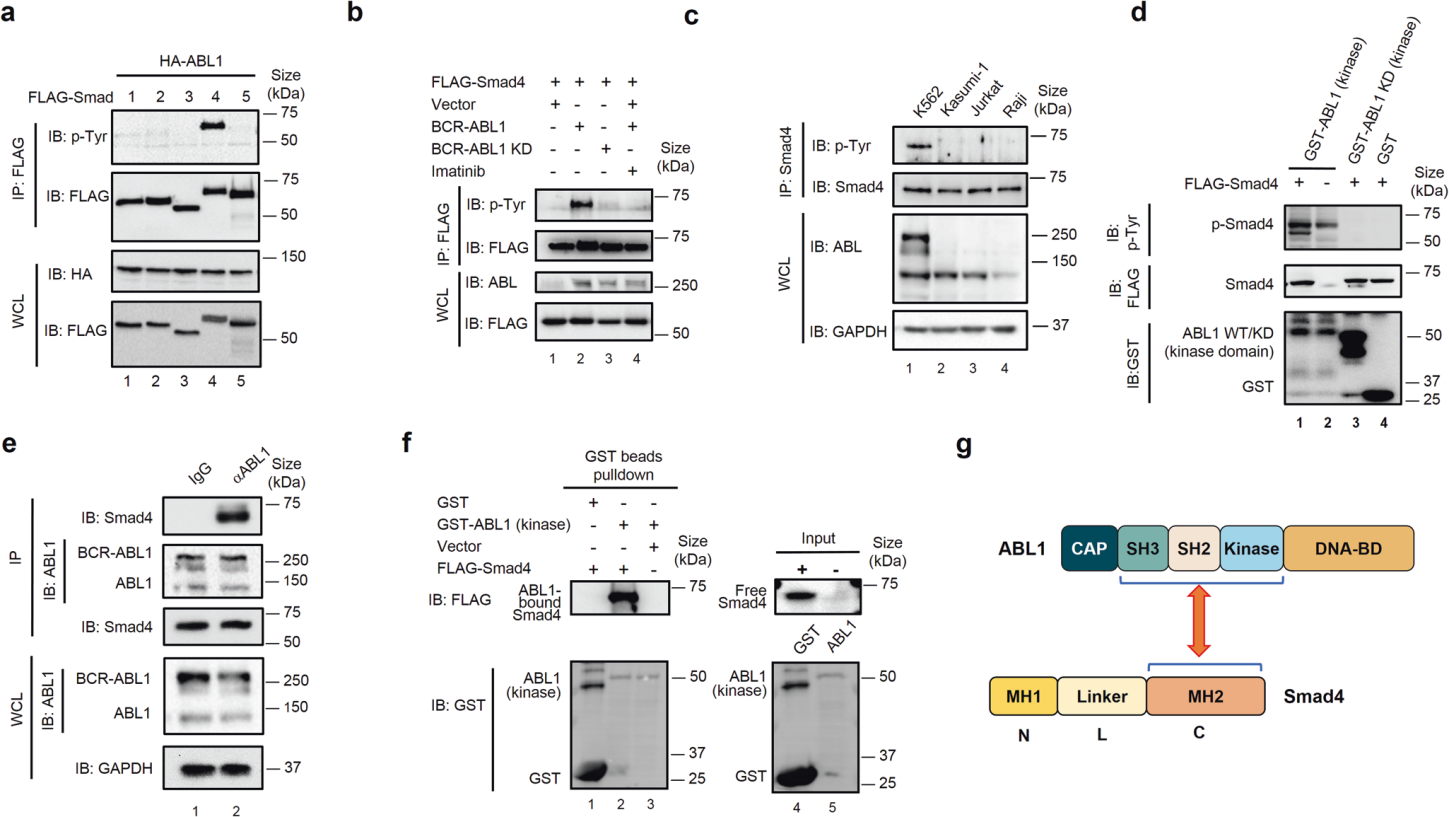
ABL1 kinases interact with and phosphorylate Smad4. **a** ABL1 specifically phosphorylates Smad4, but not R-Smads. HEK293T cells were transfected with expression plasmids encoding HA-ABL1 and FLAG-Smads. Cell lysates were harvested for immunoprepitation (IP) with anti-FLAG antibody, followed by western immunoblotting (IB) analysis with the indicated antibodies. p-Smad4 is the phosphorylated Smad4 detected by p-Tyr (PY100) – a pan-specific antibody for tyrosine phosphorylation. **b** BCR-ABL1 phosphorylates Smad4 on tyrosine residues. HEK293T cells were co-transfected with plasmids encoding FLAG-Smad4 and HA-BCR-ABL1 or HA-BCR-ABL1-KD, and treated by Imatinib (0.5 μM) as indicated. IP-western blotting was carried out as described in **a**. **c** Endogenous Smad4 is phosphorylated in BCR-ABL1-positive leukemia cells. Four leukemia cell lines were analyzed by IP-western blotting. K562: BCR-ABL1^+^ leukemia cell line; Kasumi-1, Jurkat and Raji: BCR-ABL1^-^ leukemia cell lines. **d** Recombinant ABL1 phosphorylates Smad4 on tyrosine residues in vitro. In vitro kinase reaction was carried out in a kinase reaction buffer by incubating in vitro translated FLAG-Smad4 and recombinant kinase-domain fragments of ABL1 or ABL1-KD fused to GST that were purified from *E. coli*. Smad4 phosphorylation was examined with p-Tyr antibody. **e** ABL1 interacts with Smad4 at endogenous levels. K562 cells were harvested and subjected to IP-western blotting analysis, as described in **a**, using antibodies as indicated. **f** Smad4 directly binds to the kinase-domain of ABL1. Purified GST-ABL1-kinase proteins were mixed with in vitro translated FLAG-Smad4. GST-bound proteins and input were detected by western blotting. **g** The MH2 domain of Smad4 interacts with the kinase-domain of ABL1. Schematic diagrams of ABL1 and Smad4 domains are shown, and the essential domains (pointed by the two-end arrowheads) that mediate the ABL1-Smad4 interaction are indicated

The ABL1 and BCR-ABL1 kinases phosphorylate an array of substrates involved in various cellular processes. To this end, we first tested whether ABL1 could phosphorylate Smad proteins using anti-pan-tyrosine phosphorylation antibodies. As shown in Fig. 3a, ABL1 was able to phosphorylate Smad4 on tyrosine(s) but not Smad1, Smad2, Smad3, or Smad5 (Fig. 3a). Kinase-dead mutants of ABL1 or BCR-ABL1 failed to phosphorylate Smad4. Imatinib treatment abolished Smad4 phosphorylation by ABL kinases (Fig. 3b, Supplementary Fig. 3a). Importantly, phosphorylation of endogenous Smad4 was detected in BCR-ABL1-positive K562 cells (Fig. 3c, lane 1) but not in BCR-ABL1-negative leukemia cells (lane 2–4). Moreover, we found that ABL1 directly phosphorylates Smad4. Recombinant ABL kinase, but not the ABL1-KD mutant, phosphorylated Smad4 in an in vitro kinase assay (Fig. 3d). Taken together, these results indicate that BCR-ABL1 directly phosphorylates Smad4 both in vivo and in vitro.

Since ABL1 phosphorylates Smad4 (but not other Smads), we performed co-immunoprecipitation (co-IP) experiments to determine how ABL1 binds to Smad4. When transiently expressed in HEK293T cells, Smad4 interacted with ABL1 in the reciprocal co-IP experiments (Supplementary Fig. 3b). Importantly, Smad4 and BCR-ABL1 interacted with each other at endogenous levels in K562 cells (Fig. 3e). In addition, recombinant GST-ABL1 proteins directly bound to Smad4 in a GST pull-down assay (Fig. 3f). We then examined the structural features of the ABL1-Smad4 interaction through domain mapping. When co-expressed individually with a series of deletion mutants of ABL1, Smad4 only interacted with the SH3-SH2-kinase-domain in ABL1 (Fig. 3g, Supplementary Fig. 3c, d). Conversely, the MH2 domain and the linker of Smad4 are responsible for its association with ABL1, although the interaction between ABL1 and the linker is much weaker (Fig. 3g, Supplementary Fig. 3e, f).

**Fig. 4:**
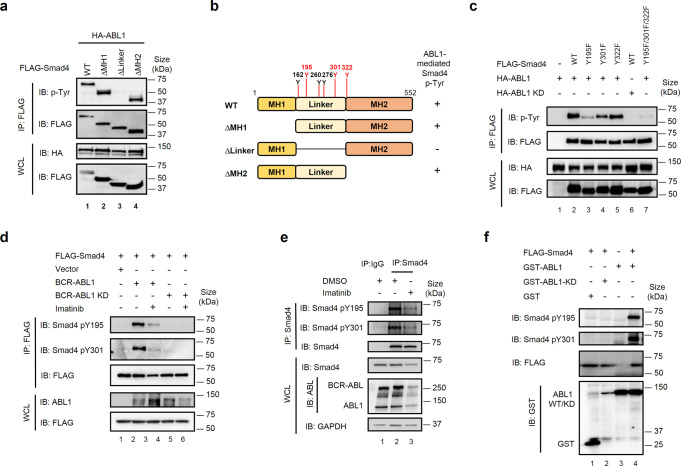
ABL1 phosphorylates Smad4 on Tyr195, Tyr301, and Tyr322. **a** ABL1 phosphorylates the linker domain of Smad4. Expression plasmids encoding HA-ABL1 and FLAG-tagged Smad4 deletion mutants were transfected into HEK293T cells. IP-western blotting was carried out as described in Fig. 3a. **b** The linker region of Smad4 contains the major phosphorylation sites. HEK293T cells were transfected with expression plasmids encoding Flag-tagged Smad4 and ABL1. Cell lysates were harvested to purify Flag-tagged Smad4 proteins via anti-FLAG IP. Affinity-purified Smad4 proteins were subjected to mass spectrometry analysis. **c** Tyrosine-to-phenylalanine (Y195F/Y301F/Y322F) substitutions abolish phosphorylation of Smad4 by ABL1. HEK293T cells were co-transfected with expression plasmids encoding FLAG- Smad4 (WT or single or triple YF mutants) and HA-ABL1 (or HA-ABL1-KD). IP-western blotting was carried out as described in Fig. 3a. **d** BCR-ABL1 phosphorylates Smad4 at Y195, Y301, and Y322. HEK293T cells were transfected with plasmids encoding BCR-ABL1 or BCR-ABL1-KD and FLAG-Smad4, and treated with Imatinib (0.5 μM). IP-western blotting was carried out as described in Fig. 3a, except that Smad4 phosphorylation was examined with the PY195 and PY301 antibody. **e** Imatinib abolishes Smad4 phosphorylation. K562 cells were treated with Imatinib (0.5 μM) and harvested for cell lysates. IP-western blotting was carried out as described in **d**. **f** Recombinant ABL1 phosphorylates Smad4 at Y195 and Y301 in vitro. In vitro kinase reaction was carried out by incubating recombinant GST-ABL1 or GST-ABL1-KD purified from *E. coil* and in vitro translated FLAG-Smad4. Smad4 phosphorylation was examined with the PY195 or PY301 antibody

We then localized the phosphorylated regions and sites of Smad4. Smad4 phosphorylation disappeared when the linker region was deleted, whereas deletion of either the MH1 or MH2 domain barely impaired phosphorylation, indicating that the phosphorylation sites were in the linker domain (Fig. 4a). Further mass spectrometry analysis revealed tyrosine residues 195 (Tyr195 or Y195), Y260, Y276, Y301, and Y322 as potential phosphorylation sites (Fig. 4b, Supplementary Fig. 4a). To identify the phosphorylation site(s), each of these tyrosine sites was substituted with phenylalanine (Y-to-F mutation). ABL1-mediated phosphorylation of Smad4 with Y195F, Y260F, Y301F, and Y322F mutations was decreased (Supplementary Fig. 4b). However, ABL kinase could not phosphorylate Y260 in the in vitro kinase assay (data not shown); therefore, Y195F, Y301F, and Y322F were selected for further studies. Although a single mutation at Y195, Y301, or Y322 only partially decreased the level of tyrosine phosphorylation, the triple mutation (Y195/Y301/Y322F) completely lost the phosphorylation signal (Fig. 4c). To further verify that these three tyrosine sites are responsible for Smad4 phosphorylation by ABL1, site-specific antibodies against pY195, pY301, or pY322 of Smad4 were generated. BCR-ABL1 and ABL1 kinases could indeed phosphorylate these three sites, as confirmed using specific pY195 and pY301 antibodies (Fig. 4d and Supplementary Fig. 4c). Moreover, site-specific phosphorylation of endogenous Smad4 was detected in K562 cells, and Imatinib diminished this phosphorylation (Fig. 4e). Additionally, ABL1 directly phosphorylated Smad4 at Y195 and Y301 in an in vitro kinase assay (Fig. 4f). Overall, this evidence supports the notion that ABL kinases directly phosphorylate Smad4 on tyrosine residues 195, 301, and 322.

### Phosphorylation of Smad4 impairs its interaction with transcription co-activator p300

**Fig. 5:**
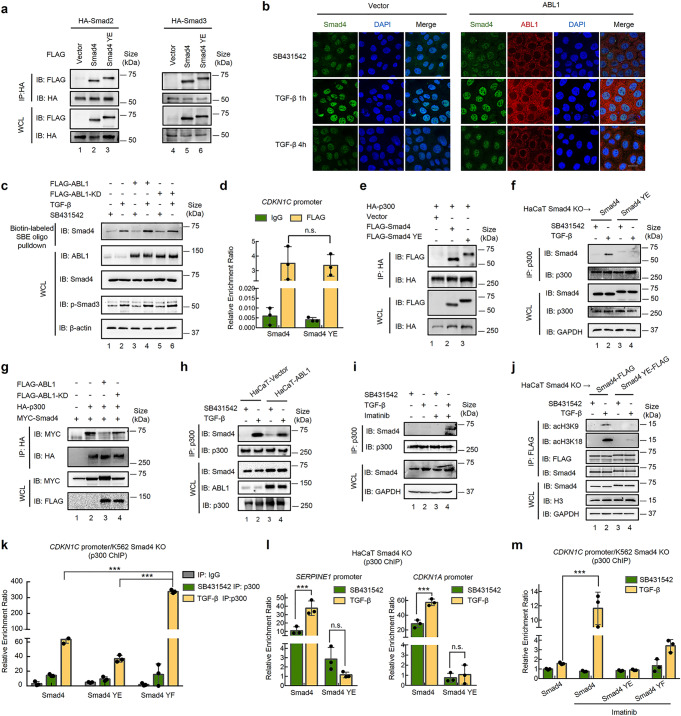
Phosphorylation of Smad4 on Y195/Y301/Y322 blocks its interaction with p300. **a** Phosphorylation of Smad4 at Y195/Y301/Y322 does not affect the assembly of the Smad2-Smad4 complex or the Smad3-Smad4 complex. Plasmids encoding HA-Smad2 or HA-Smad3 and FLAG-Smad4 or FLAG-Smad4 YE mutant were transfected into HEK293T cells. IP-western blotting was carried out as described in Fig. 3a. **b** ABL1 has no effects on the nuclear localization of Smad4. HaCaT cells stably expressing ABL1 were treated with TGF-β (2 ng/ml) and subjected to immunofluorescence with the indicated antibodies. Red, immunostained ABL1; green, immunostained Smad4; purple, 4′,6-diamidino-2-phenylindole (DAPI) nuclear staining. Scale bars, 20 μm. **c** Ectopic expression of ABL1 does not affect DNA binding of Smad4. HaCaT cells were transfected with plasmids encoding ABL1 or ABL1-KD and treated with TGF-β (2 ng/ml). Cell lysates were collected, incubated with biotin-labeled SBE probes and immobilized on streptavidin beads. Retrieved proteins were determined by western blotting with indicated antibodies. **d** Triple Y195E/Y301E/Y322E (YE) mutations do not affect Smad4 binding to the *CDKN1C* promoter. HEK293T cells were transfected with expression plasmids expressing FLAG-Smad4 or FLAG-Smad4 YE mutant and treated with TGF-β for 2 h. Chromatin immunoprecipitation (ChIP) was carried out with anti-FLAG antibody and qPCR analysis using primers specific to *CDKN1C*. Data are shown as mean ± SEM; *n* = 3. Statistical analysis was performed using ANOVA. n.s. represents no significance. **e** YE mutations attenuate the interaction of Smad4 with the transcription co-activator p300. HEK293T cells were transfected with expression plasmids encoding HA-p300 and FLAG-Smad4 or FLAG-Smad4-YE mutant. IP-western blotting was carried out as described in Fig. 3a. **f** YE mutations abolish the endogenous interaction between Smad4 and p300. Smad4−/− HaCaT cells stably expressing WT or YE mutant of Smad4 were treated with TGF-β or SB431542. IP-western blotting was carried out as described in Fig. 3a. **g** ABL1 attenuates the Smad4-p300 interaction. HEK293T cells were transfected with expression plasmids encoding HA-p300, FLAG-ABL1 or FLAG-ABL1-KD, and MYC-Smad4. IP-western blotting was carried out as described in Fig. 3a. **h** ABL1 attenuates the endogenous Smad4-p300 interaction. HaCaT cells stably expressing ABL1 were treated with TGF-β or SB431542. IP-western blotting was carried out as described in Fig. 3a. **i** Imatinib enhances the endogenous Smad4-p300 interaction. K562 cells were treated with TGF-β (2 ng/ml), SB431542 (5 μM), or Imatinib (0.5 μM) as indicated. IP-western blotting was carried out as described in Fig. 3a. **j** The YE mutant of Smad4 loses the ability to interact with acetylated Histone H3. Smad4−/− 0HaCaT cells stably expressing WT or the YE mutant of Smad4 were treated with TGF-β or SB431542. IP-western blotting was carried out as described in Fig. 3a. **k** The YF mutant of Smad4 promotes the p300 occupancy on the *CDKN1C* promoter. Smad4−/−K562 cells stably expressing WT, YE, or YF mutant of Smad4 were treated with TGF-β or SB431542. ChIP was carried out as described in Fig. 5d by using anti-p300 antibody. Data are shown as mean ± SEM; *n* = 3. Statistical analysis was performed using ANOVA. ***P < 0.001. **l** The YE mutant of Smad4 abolishes the p300 occupancy on the *SERPINE1* and *CDKN1A* promoter. Smad4−/− HaCaT cells stably expressing WT or YE mutant of Smad4 were treated with TGF-β (4 ng/ml) or SB431542. Anti-p300 ChIP and qPCR analysis were similarly carried out, as described in **k**, using primers specific to *SERPINE1* (left) and *CDKN1A* (right). Data are shown as mean ± SEM; *n* = 3. Statistical analysis was performed using ANOVA. ***P < 0.001. n.s. represents no significance. **m** Imatinib markedly enhanced TGF-β-induced p300 binding to the *CDKN1C* promoter (~12-fold increase) dependent of Smad4. Smad4−/− K562 cells stably expressing WT, YE, or YF mutant of Smad4 were treated with TGF-β (2 ng/ml) or SB431542 and/or Imatinib. Anti-p300 ChIP and qPCR were done as described in Fig. **k**. Data are shown as mean ± SEM; *n* = 3. Statistical analysis was performed using ANOVA. ***P < 0.001

To decipher the mechanisms by which ABL-induced phosphorylation of Smad4 impairs TGF-β signaling, we assessed the impact of ABL/Smad4 phosphorylation on the signaling steps, from Smad activation to their transcription activities, in the canonical TGF-β pathway. We found that neither BCR-ABL1 knockdown nor ABL1 overexpression affected the phosphorylation levels of Smad2 and Smad3 in K562 cells (Supplementary Fig. 5a). The triple phosphorylation-mimicking Y195E/Y301E/Y322E (YE) mutant of Smad4 exhibited a similar binding capacity with Smad3 or Smad2 to that of wild-type Smad4, apparently without any effects on the assembly of the Smad2-Smad4 and Smad3-Smad4 complexes (Fig. 5a). There was also no alteration in the interaction between Smad3 and Smad4 in HaCaT cells stably expressing ABL1 (Supplementary Fig. 5b). Therefore, we investigated whether Smad4 phosphorylation affected its nuclear translocation. First, ABL1 phosphorylated Smad4 mainly in the cytoplasm, as the nuclear-localized Smad4 NES mutant failed to be phosphorylated (Supplementary Fig. 5c), which is consistent with the cytoplasmic localization of ABL1 (Supplementary Fig. 5d). In addition, ABL1 overexpression did not affect the nuclear localization of either total Smad4 (Fig. 5b) or phosphorylated Smad4 (Supplementary Fig. 5d). Since Smad4 has direct DNA-binding activity on SBE regions,^24^ we assessed whether Smad4 phosphorylation affects its DNA-binding ability. We found that ABL1 or ABL1-KD had minor effects on Smad4 binding to biotin-labeled SBEs (Fig. 5c), and the YE mutation did not alter the SBE-binding activity of Smad4 (Supplementary Fig. 5e). Moreover, we used ChIP-qPCR analysis to determine the occupancy of Smad4 and the YE mutant in the promoters of TGF-β target genes. The results showed that the YE mutation did not affect its occupancy of the promoters of *CDKN1C* (Fig. 5d), *CDKN2B*, and *SERPINE1* in response to TGF-β (Supplementary Fig. 5g), further confirming that Smad4 phosphorylation does not influence its DNA-binding activity under physiological conditions.

Smad4 has direct DNA-binding activity on SBEs and transactivation activity through its interaction with p300/CBP, a critical transcription co-activator in TGF-β signaling.^29^ As Y301 and Y322 are located in the Smad4 activation domain (SAD), a proline-rich transcriptional activation domain that binds p300,^29^ we speculated that tyrosine phosphorylation might alter the ability of Smad4 to bind p300. Indeed, co-IP assays showed that compared to wild-type Smad4, the Smad4 YE mutant had a reduced ability to interact with p300 (Fig. 5e) or CBP (Supplementary Fig. 5h), which is the ortholog of p300.^30^ The failure of the YE mutant to bind p300 was more obvious in Smad4-null HaCaT cells (Fig. 5f). Conversely, the YF mutant had a stronger interaction between p300 (Supplementary Fig. 5i). Consistently, overexpression of ABL1, but not ABL1-KD, reduced the Smad4-p300 interaction (Fig. 5g). Forced expression of ABL1 also decreased the endogenous Smad4-p300 interaction in HaCaT cells (Fig. 5h). In K562 cells, the endogenous interaction between Smad4 and p300 was weak and dramatically enhanced after Imatinib treatment (Fig. 5i). Furthermore, the YE mutant lost the ability to associate with acetylated histone H3, a major epigenetic target of p300^8,31^ (Fig. 5j). Finally, anti-p300 ChIP assays were performed to determine how Smad4 phosphorylation influences the p300 binding to chromatin on TGF-β-responsive promoters. In Smad4−/− K562 cells, the re-introduction of Smad4 moderately restored p300 binding to chromatin in response to TGF-β, whereas the phospho-mimicking YE mutant only weakly increased p300 binding to chromatin. However, the non-phosphorylatable YF mutant dramatically promoted p300 occupancy of the *CDKN1C* promoter (Fig. 5k). Hence, we concluded that BCR-ABL1 prevents TGF-β-dependent p300 binding to chromatin, which is dependent on the phosphorylation status of Smad4. This notion was further supported by experiments performed in Smad4−/− HaCaT cells, in which ABL1 was not activated. Re-introduction of Smad4 enabled the TGF-β-induced recruitment of p300 proteins onto the promoter region of *SERPINE1* and *CDKN1A*; in sharp contrast, the chromatin occupancy of p300 on these promoters in response to TGF-β was absent in Smad4−/− HaCaT cells re-expressing the YE mutant (Fig. 5l). Moreover, Imatinib significantly enhanced TGF-β-induced p300 binding to the CDKN1C promoter, and this enhancement occurred in the presence of WT Smad4 but not Smad4 YE (Fig. 5m), further supporting the notion that Imatinib enhanced TGF-β responses through blocking negative phosphorylation on Smad4. In conclusion, ABL1-mediated phosphorylation of Smad4 impairs its association with p300 and subsequent p300 deposition on the promoters of TGF-β target genes.

### Tyrosine-phosphorylated Smad4 loses its growth inhibitory function

**Fig. 6:**
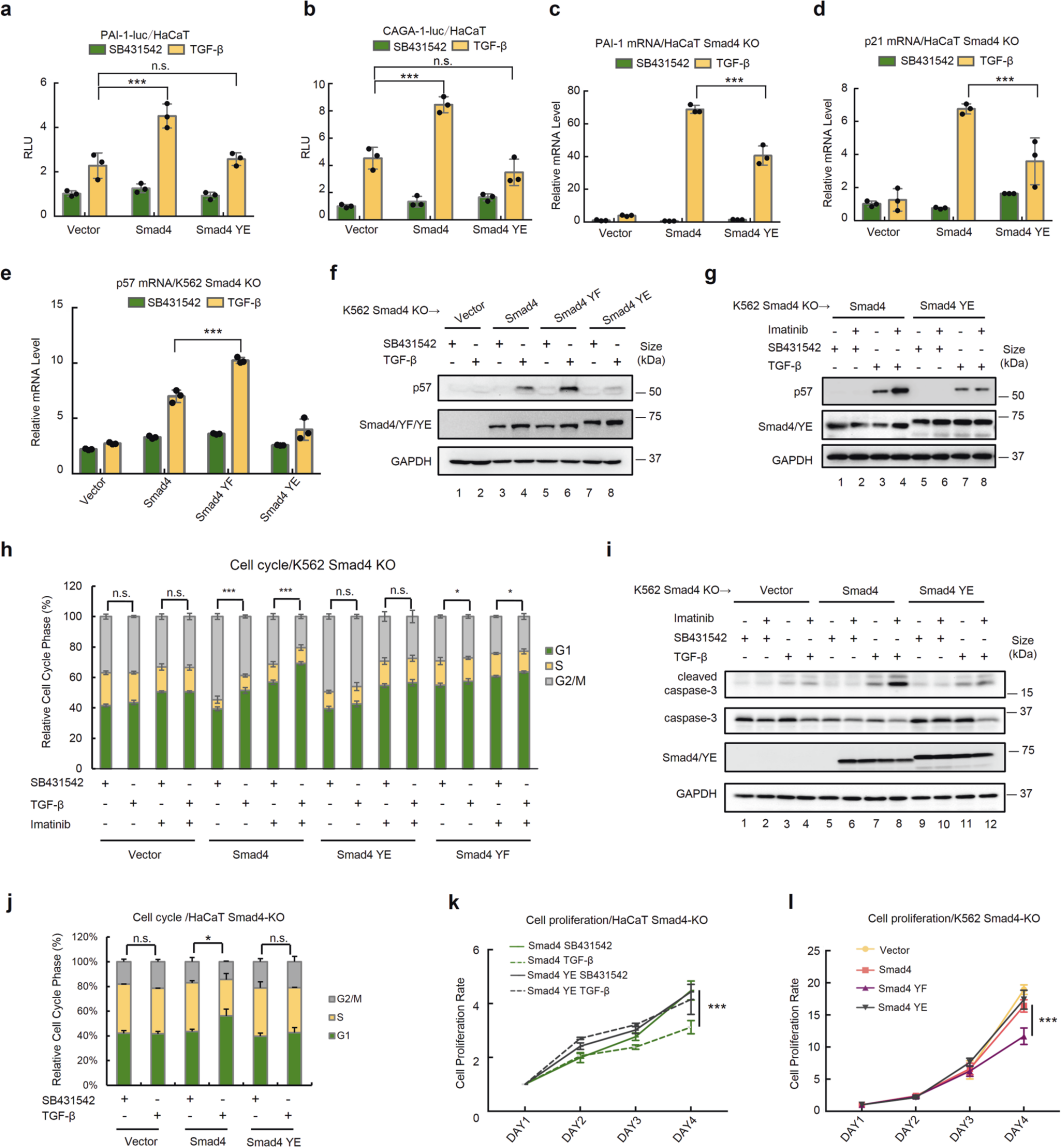
Smad4 tyrosine phosphorylation impairs its cell growth inhibitory effect. **a**, **b** The YE mutant of Smad4 loses its ability to enhance TGF-β-induced transcriptional responses. HaCaT cell transfection, TGF-β treatment, and reporter assays were carried out as described in Fig. 1a. PAI-1-luc (**a**) or CAGA-luc (**b**) reporter gene were used. Data are shown as mean ± SEM; *n* = 3. Statistical analysis was performed using ANOVA. ***P < 0.001. n.s. represents no significance. **c**, **d** The Smad4 YE mutation attenuates TGF-β-induced transcription. Smad4−/− HaCaT cells stably expressing Smad4 or Smad4 YE were treated with TGF-β (2 ng/ml) or SB431542 for 12 h. Total RNAs were extracted and subjected to qRT-PCR analysis using primers specific to PAI-1 (**c**) and p21 (**d**) as described in Fig. 1b. Data are shown as mean ± SEM; *n* = 3. Statistical analysis was performed using ANOVA. ***P < 0.001. **e**, **f** The Smad4 YF mutant gains a stronger ability to induce p57 expression. Smad4−/− K562 cells stably expressing Smad4, Smad4 YF, or Smad4 YE were treated with TGF-β (2 ng/ml) or SB431542. **e** Total RNAs were extracted and subjected to qRT-PCR analysis, as described in Fig. 1b, using primers specific to p57. Data are shown as mean ± SEM; *n* = 3. Statistical analysis was performed using ANOVA. ***P < 0.001. **f** anti-p57 western blotting was done as described in Fig. 1h. **g** Imatinib fails to enhance p57 expression in Smad4 YE-expressing K562 cells. Smad4−/− K562 cells stably expressing Smad4 or the YE mutant were treated with TGF-β, SB431542, and/or Imatinib as indicated for 24 h. Western blotting was done with appropriate antibodies as indicated. **h** The YF mutation promotes TGF-β-induced cell cycle arrest. Smad4−/− K562 cells stably expressing Smad4 or the YE mutant or the YF mutant were treated with TGF-β or SB431542. FACS analysis was done as described in Fig. 1i. Statistical analysis showing cells in G1 stage was performed using ANOVA. ***P < 0.001. **i** Smad4 YE mutant fails to restore TGF-β-induced cleavage of caspase-3. Cell treatment, lysate preparation, and western blotting were carried out, as described in Fig. 1d, with appropriate antibodies as indicated. **j** Re-expression of the YE mutant in Smad4−/− HaCaT cells fails to restore TGF-β-induced G1 arrest. Smad4−/− HaCaT cells stably expressing Smad4 or Smad4 YE were treated TGF-β (2 ng/ml, +TGF-β) or SB431542 (−TGF-β). Statistical analysis showing cells in G1 stage was performed using ANOVA. ***P < 0.001. **k** Re-expression of the YE mutant in Smad4−/− HaCaT cells fails to restore TGF-β-induced suppression of proliferation. Smad4−/− HaCaT cells stably expressing Smad4 or Smad4 YE were treated with TGF-β (2 ng/ml) or SB431542 (5 μM) for indicated days. Cells were analyzed for cell proliferation by using CCK8 assay. Data are shown as mean ± SEM; *n* = 3. Statistical analysis was performed using ANOVA. ***P < 0.001. **l** The Smad4 YF mutant is capable to inhibit the proliferation of K562 cells in the absence of exogenous TGF-β. Smad4−/− K562 cells stably expressing Smad4, Smad4 YF, or YE were analyzed for cell proliferation by using CCK8 assay. Data are shown as mean ± SEM; *n* = 3. Statistical analysis was performed using ANOVA. ***P < 0.001

In agreement with the observation that Smad4 phosphorylation interfered with its binding to p300/CBP on chromatin, the phospho-mimicking YE mutant lost the ability to induce both PAI-luc and CAGA-luc reporter responses in HaCaT cells. As a positive control, Smad4 significantly enhanced these reporter responses (Fig. 6a, b). Accordingly, Smad4-rescued TGF-β-induced expression of PAI-1 and p21 in Smad4−/− HaCaT cells; The YE mutant exhibited only partial rescue effect compared to wildtype Smad4 (Fig. 6c, d). In Smad4−/− K562 cells, the YE mutant was unable to rescue TGF-β-induced expression of p57, whereas the non-phosphorylatable YF mutant conferred even more robust induction of p57 expression (Fig. 6e, f). Notably, Imatinib further enhanced TGF-β-induced p57 expression in Smad4-rescued Smad4−/− K562 cells but not in YE mutant-rescued cells (Fig. 6g and Supplementary Fig. 6a, b). Similar to wild-type Smad4, each of the single-point mutants of Smad4 on the three individual sites (both YE and YF) retained the ability to rescue TGF-β-induced p57 expression in Smad4−/− K562 cells (Supplementary Fig. 6c–e), indicating that the collective actions of the three phosphorylation sites suppress Smad4-mediated TGF-β signaling.

Next, we assessed the influence of Smad4 phosphorylation on the physiological functions of TGF-β. In Smad4−/− K562 cells that had no TGF-β activity, re-introduction of the YF mutant of Smad4 showed a growth inhibitory response that was equal to or greater than that of wild-type Smad4-rescued cells, whereas the YE- and vector-rescued cells did not respond to TGF-β (Fig. 6h). Meanwhile, Smad4 restored TGF-β-induced apoptosis, as indicated by the increased cleavage of caspase-3, especially in the presence of Imatinib (Fig. 6i), whereas the YE mutant failed to restore the same response in K562 cells (Fig. 6i and Supplementary Fig. 6f). In HaCaT cells, Smad4 depletion blocked the TGF-β-induced cell cycle arrest. Re-expression of wild-type Smad4, but not of the YE mutant, restored TGF-β-induced G1 arrest and growth inhibition (Fig. 6j, k). Moreover, the YF mutant of Smad4 potently suppressed the proliferation of K562 cells, even in the absence of TGF-β (Fig. 6l). In summary, these results indicate that phosphorylation of Smad4 at Y195/Y301/Y322 sites suppresses TGF-β cytostatic actions.

### Tyrosine-phosphorylated Smad4 promoted leukemogenesis in vivo

**Fig. 7:**
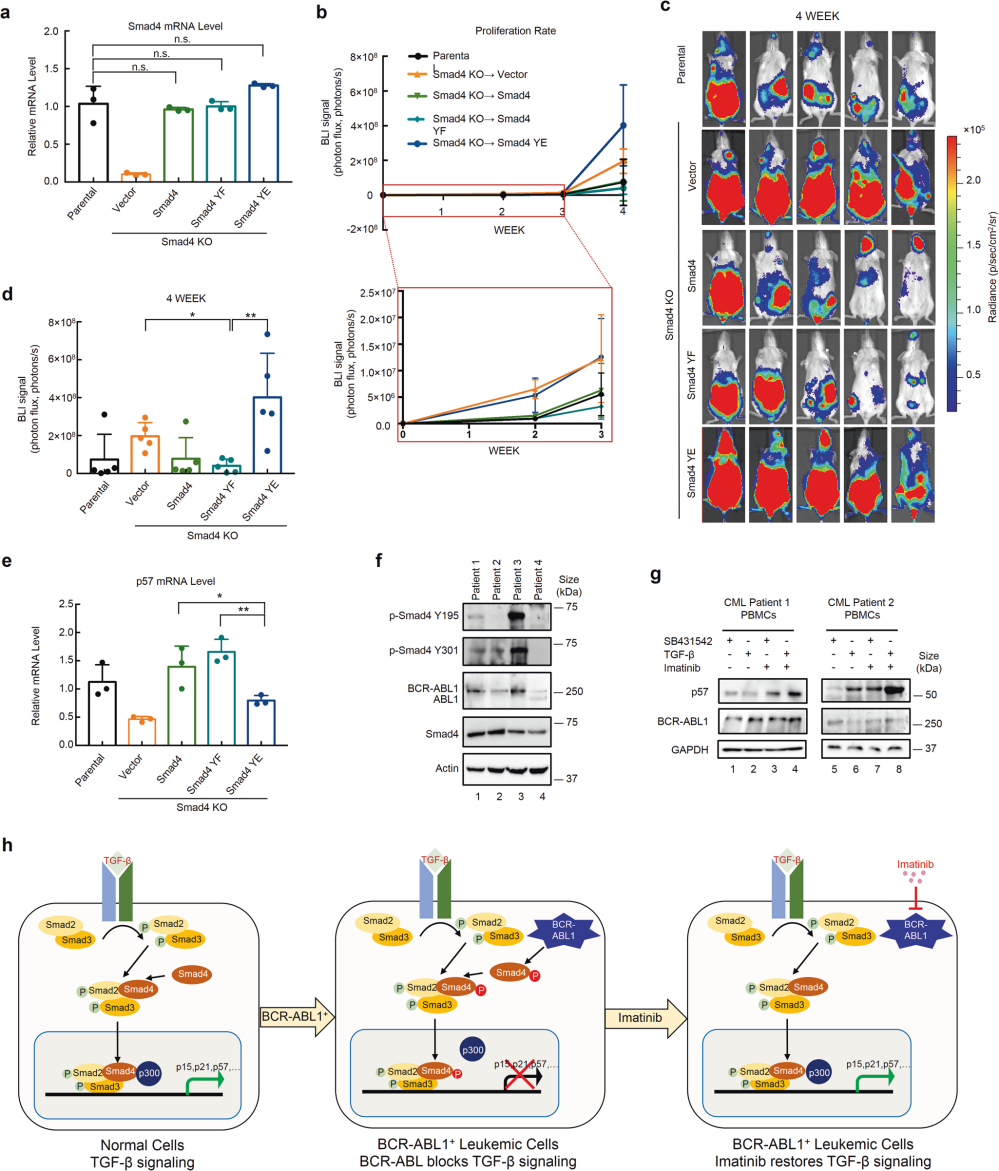
Tyrosine phosphorylation disables the tumor-suppressing activity of Smad4 in vivo. **a** Smad4 variants (WT/YF/YE) are re-expressed at similar levels in Smad4−/− K562 cells. Smad4−/− K562 cells stably expressing Smad4 or its YE/YF mutants were harvested for total RNA extraction and subsequent qRT-PCR analysis using primers specific to Smad4. Statistical analysis was performed using ANOVA. n.s. represents no significance. **b** Phospho-mimicking YE mutations disable the tumor-suppressing activity of Smad4 in mice. 8-week-old NSG female mice were irradiated with 1.5 Gy X-ray and then transplanted with 4 × 10^6^ luciferase-expressing K562 cells via tail vein injection. Mice were placed in the IVIS SpectrumCT In Vivo Imaging System for bioluminescence (BLI) measurement every week after injection. BLI signals indicating proliferation rates of K562 cells expressing Smad4 or its variant are plotted over weeks. BLI signals in the first 3 weeks are shown in the enlarged box for easy visualization. **c** Mice carrying the YE mutant-expressing K562 cells develop more severe leukemia. BLI images were taken from representative mice of each group at week 4 after being injected with Smad4−/− K562 cells stably expressing Smad4 or its YE/YF mutant. **d** Statistical analysis of BLI quantitation in **c**. **e** The YE mutant fails to induce p57 mRNA in leukemia. K562-derived leukemia cells were isolated from mice expressing Smad4 or its YE/YF variant and subjected to qRT-PCR analysis using primers specific to p57. Data are shown as mean ± SEM; *n* = 3. Statistical analysis was performed using ANOVA. *P < 0.05. ***P < 0.001. **f** Tyrosine phosphorylation of Smad4 is correlated with BCR-ABL1. Bone marrows from three CML patients (lane 1–3) and blood samples from patient 4 (lane 4) were prepared and subjected to western blotting analysis with indicated antibodies. **g** Imatinib enhances TGF-β-induced p57 expression in CML patient PBMCs. PBMCs collected from CML patients were treated with TGF-β (2 ng/ml), SB431542 (5 μM), and/or Imatinib (0.5 μM) for 24 h. Cell lysates were prepared and subjected to western blotting analysis with antibodies as indicated. **h** A working model for the effect of tyrosine phosphorylation of Smad4 in TGF-β signaling in oncogenic ABL1-positive tumors. In tumors expressing constitutively active BCR-ABL1, Smad4 is phosphorylated on tyrosines. Linker-phosphorylated Smad4 loses its ability to interact with the co-activator p300 and fails to induce the expression of genes necessary for tumor suppression. ABL inhibitor Imatinib potently restores TGF-β tumor-suppressive signaling in BCR-ABL1-positive leukemia

We extended our study to investigate whether the tyrosine phosphorylation of Smad4 influences leukemogenesis in a mouse model. NSG mice were exposed to a median lethal dose of radiation to deplete their immune system, and were then injected with K562 cells that stably expressed a Smad4 variant at a level comparable to that in the parental cells (Fig. 7a). K562 cells also expressed firefly luciferase to facilitate bioluminescence detection in vivo. Notably, the expression of different Smad4 variants in K562 cells resulted in different growth rates (Fig. 7b). The majority of mice showed varying degrees of leukemia phenotypes after 3 (Supplementary Fig. 7a, b) or 4 weeks (Fig. 7c, d). Notably, the cells harboring the YE mutant showed the highest leukemia burden, whereas the YF mutant showed the lowest (Fig. 7a, c, d and Supplementary Fig. 7a, b), which is well correlated with the loss or gain of the tumor suppressor role in the YE and YF mutants, respectively. Consistent with this, expression of p57 was significantly decreased in Smad4−/− leukemic cells isolated from mice (Fig. 7e); however, the p57 level was restored in leukemic cells with re-introduction of Smad4 and its YF mutant. YE mutant expression had little effect on rescuing p57 expression in the leukemia samples examined.

Moreover, we examined the phosphorylation level of Smad4 in BCR-ABL1-positive human CML patients. Proteins extracted from bone marrows of three CML patients (patients 1–3) and peripheral blood of one CML patients (patient 4) were subjected to western blotting analysis (Fig. 7f). Our results showed a clear correlation between p-Smad4 level and BCR-ABL1 expression. Furthermore, PMBCs were isolated from the first two CML patients, of which PBMCs from Patient 1 expressed a higher level of BCR-ABL1 than Patient 2 (Fig. 7f, g), and were subjected to assessing TGF-β-induced p57 expression. In Patient 1 PBMCs that had a high level of BCR-ABL1, TGF-β treatment did not induce p57, whereas Imatinib could restore the TGF-β-induced p57 expression (Fig. 7g). In Patient 2 PBMCs with a low level of BCR-ABL1, TGF-β could induce p57 expression and Imatinib further boosted the TGF-β response (Fig. 7g). These results suggest that Imatinib also sensitized BCR-ABL1-positive cells to TGF-β in human PBMCs.

## Discussion

Loss of the antiproliferative response is a hallmark of human cancers. TGF-β is one such cytokine that suppresses cell growth. Tumor cells have developed a number of strategies to escape from TGF-β anti-growth control. One major mechanism to resist the cytostatic effect of TGF-β is through frequent inactivating mutations and deletions in Smad4/DPC4 in gastrointestinal and pancreatic cancer.^32^ Although a few mutations or deletions of Smad4 have been reported to attenuate TGF-β-mediated signaling in AML, genetic mutations in Smad4 are rare events in leukemia. Nonetheless, several lines of evidence have demonstrated that TGF-β signaling is dysregulated in leukemia.^33,34^ Here, we report that insensitivity to TGF-β in CML comes from the BCR-ABL1 oncoprotein that disrupts the homeostatic function of TGF-β signaling. Mechanistically, BCR-ABL1 phosphorylates Smad4 to block its interaction with critical transcriptional co-activators p300/CBP, impeding TGF-β transcriptional responses. Conversely, Imatinib treatment abolished the BCR-ABL-mediated phosphorylation of Smad4, and consequently restored the TGF-β cytostatic response.

Despite being discovered more than 25 years ago, Smad proteins have rarely been reported to be phosphorylated on tyrosine residues. In this study, our biochemical evidence suggests that Smad4 is indeed phosphorylated by BCR-ABL1, and phosphorylation occurs at tyrosine residues 195, 301, and 322 (Y195/Y301/Y322) in the linker region of Smad4. First, combined approaches using mass spectrometry and co-IP experiments revealed that Y195/Y301/Y322 of Smad4 are the major phosphorylation sites in the presence of ABL1 kinases. Secondly, by using the site mutagenesis approach, we found that the triple Y-to-F substitutions (i.e., Y195F/Y301F/Y322F mutants) completely lost phosphorylation by ABL1 kinases. Thirdly, we generated site-specific phosphorylation antibodies against each of pY195, pY301, or pY322 of Smad4, further confirming that BCR-ABL1 and ABL1 kinases could indeed phosphorylate these three sites in vitro and in vivo.

Our biochemical studies on the BCR-ABL1-Smad4 interplay have significant implications for human leukemogenesis. We detected phosphorylation of Smad4 (Y195/Y301) in BCR-ABL1 patients samples (bone marrow or blood). Although we have only tested limited number of human CML patients, phosphorylation of Smad4 Y195 and Y301 exists in patients. Importantly, the level of p-Smad4 correlates to that of BCR-ABL1 protein. Therefore, phospho-Y195/Y301/Y322 may be optimized as a novel biomarker for BCR-ABL1-positive tumors. In addition to CML, approximately 25–30% of adult B-cell ALL cases^35^ and a small percentage of AML cases^28^ are BCR-ABL1-positive. Thus, the effects of BCR-ABL1 on TGF-β/Smad4 signaling may also exist in other BCR-ABL1-positive leukemia cells. Further investigation of Smad4 tyrosine phosphorylation and its physiological impact on other kinds of leukemia will reveal whether Smad4 tyrosine phosphorylation is a specific biomarker for different kinds of leukemia. Moreover, it is plausible that loss of Smad4 phosphorylation may also contribute to TKI resistance in CML patients with TKI intolerance.

Smad4 is a common signal transducer and transcriptional co-activator in TGF-β signaling. How does tyrosine phosphorylation influence the activity of Smad4? The Y195/Y301/Y322 sites are also in the linker region, and interestingly, Y301/Y322 are in the p300/CBP-interacting SAD domain.^8,29^ Notably, the triple Y-to-E phosphorylation-mimicking substitutions indeed disrupt the p300/CBP-Smad4 interaction, whereas the triple Y-to-F mutants had little effects. Neither Y-to-E nor Y-to-F mutants affect the Smad complex formation and the Smad-chromatin interaction. Yet, tyrosine phosphorylation-triggered loss of p300/CBP binding suffices to abrogate the transcriptional activity of Smad4. Therefore, tyrosine phosphorylation of Smad4 at Y195/Y301/Y322 sites by ABL1 renders Smad4 dysfunctional and consequently blocks expression of CDK inhibitors and other growth regulators that are strictly controlled by TGF-β signaling, thus contributing to uncontrolled proliferation of leukemia cells. This investigation explains in detail how TGF-β anti-growth signaling is blocked in BCR-ABL1-positive CML, and for the first time identified the direct impact of oncoproteins on the TGF-β signaling pathway in leukemia.

Thus, BCR-ABL1 suffices to shut down canonical TGF-β signaling in CML even in the absence of Smad4 deletions or inactivating mutations. This ABL1-Smad4 interplay agrees with previous reports that loss of TGF-β responses can be acquired by the oncogenic activation of transcription factors that TGF-β signaling cross-talks with,^36–41^ and further extends to the direct antagonism of TGF-β cytostatic signaling by another oncogenic tyrosine kinase. We recently reported that Smad4 tyrosine phosphorylation also causes TGF-β resistance in ALK-positive tumors such as lung tumors, lymphoma, and neuroblastoma.^42^ Thus, the inhibitory phosphorylation of Smad4 may represent a common mechanism for blocking TGF-β anti-growth functions. However, Smad4 phosphorylation by ALK^42^ or BCR-ABL1 (this study) differs in biochemical properties and signaling outcomes. BCR-ABL1 and ALK phosphorylate Smad4 at different tyrosine residues in different regions. BCR-ABL1 phosphorylates Smad4 in the p300/CBP-binding region, thereby blocking Smad4 transactivation, whereas ALK targets the DNA-binding domain of Smad4, therefore blocking the DNA-binding activity of Smad4. Based on these findings, it is conceivable that as a central signal transducer, the tumor suppressor activities of Smad4 can be antagonized by oncogenic kinases through tyrosine phoshphoryaltion, thus serving as a major mechanism that favors tumorigenesis (Fig. 7h).^36^ Since a high proportion of tyrosine kinases account for more than half of the proto-oncogenes, whether more kinases can phosphorylate Smad4 or other Smad family members in cancer is also worth further exploration.

Imatinib is an effective drug for treating the majority of CML patients, which is mostly attributed to the blockade of the known growth-promoting properties of BCR-ABL1, such as activation of the downstream MAPK, AKT, and STAT pathways. Our study expands the substrate scope of BCR-ABL1 to the tumor suppressor Smad4, and thus adds another layer of complexity to Imatinib effects. This study has several important clinical implications. Essentially, Imatinib blocks the inhibitory tyrosine phosphorylation of Smad4, thereby sensitizing CML cells to achieve a better response to TGF-β. Thus, by inhibiting the tyrosine kinase activity of ABL1, Imatinib enhances TGF-β-mediated growth inhibition and apoptosis in CML cells, which is beneficial for patients with CML. Second, along the same line, the observation that combination treatment of cultured CML cells with Imatinib and TGF-β produced better growth inhibitory and apoptotic effects suggests a possible combination therapy involving TKI and TGF-β agonists. A subgroup of patients is known to have poor responses to TKI therapy or to develop resistance to treatment in a longer follow-up.^43^ Our study implies an interesting possibility that the combination of Imatinib and TGF-β agonists is a potential alternative therapy for CML. Finally, it remains unknown whether enhanced TGF-β responses could be accompanied by harmful responses, as TGF-β-induced EMT-like effects may cause drug resistance.^44^ The answers to these questions require further investigation.

Overall, in this study, we revealed that Smad4 phosphorylation by BCR-ABL1 plays an essential role in CML, which adds a novel mechanism to explain how TGF-β signaling is dysregulated in leukemia. Above all, these results further suggest a promising combined Imatinib therapeutic strategy with fewer severe side effects and better clinical efficacy, especially in Imatinib-resistant patients.

## Materials and methods

### Cell proliferation and cell cycle analysis

Cell proliferation was measured using a CCK8 kit, according to the manufacturer’s instructions (Vazyme, Nanjing, China). Briefly, cells were split into 96‐well plates and 10 µL of the CCK‐8 solution was added to each well of the microplate. After being placed in a humidified incubator for 2 h, the absorbance at 450 nm was measured by using a microplate reader. Cell proliferation rate represents the fold change from Day 0. For cell cycle analysis using flow cytometry, the cells were seeded in a six-well plate at a density of 100,000 cells/ml. After 24 h, the cells were harvested, fixed overnight using 70% cold ethanol, and stained using PI/RNase Staining Buffer according to the manufacturer’s instructions (MultiSciences Biotech, Hangzhou, China). Cell cycle profiles were measured using a Beckman CytoFlex instrument, and the percentages of different cell cycle phases were analyzed using FlowJo software. The sub-G1 peak in the cell cycle distribution analysis indicates dead cells.

### In vitro protein phosphorylation and interaction assays

Recombinant Flag-Smad4 and its mutants were obtained using in vitro TNT® Quick Coupled Transcription/Translation System (Promega, WI, US). Recombinant GST-ABL1 kinase truncation (SH3-SH2-kinase-domain) and its kinase-dead mutant were purified from *E. coli* BL21 (DE3) strain. In a typical phosphorylation reaction, 3 μg Flag-Smad4 and 1 μg ABL1 kinase were mixed in 50 μl kinase reaction buffer (50 mM Tris-HCl [pH7.5], 5 mM MgCl_2_, and 30 μM ATP) at 37 °C for 0.5 h. Smad4 phosphorylation was analyzed by western blotting using PY100 or a phospho-specific antibody.

In a GST pull-down assay to test the ABL-Smad4 interaction in vitro, the experimental procedure was performed as previously described.^24^ Briefly, recombinant Flag-Smad4 (or its mutants) was incubated with recombinant GST-ABL1 on GST beads (GE Healthcare, MA, US). ABL1-bound Smad4 proteins were analyzed by anti-FLAG western blotting.

### Mass spectrometry analysis

Flag-Smad4 and ABL1 plasmids were transfected into the HEK293T cells. Smad4 proteins were purified and enriched on anti-FLAG antibody-conjugated Sepharose beads (Sigma-Aldrich, MO, US). Denatured protein samples were loaded and separated on an SDS-PAGE gel, which was then stained with Coomassie Blue. The Flag-Smad4 protein band was cut and digested with chymotrypsin, Glu-c, and trypsin. LC-MS/MS was performed and the detailed phosphorylation sites were analyzed as described before.^42^

### Leukemia xenograft model

Female NSG mice were purchased from Shanghai Research Center for Model Organisms. 8-week-old NSG mice were irradiated (1.5 Gy) and injected with K562-luciferase cells^45^ (4 × 10^6^) via the tail vein. Leukemia engraftments were measured using the IVIS SpectrumCT In Vivo Imaging System (Perkin Elmer, MA, US).

All of the procedures involving animal experiments were approved by the Institutional Animal Care and Use Committee (IACUC) at Zhejiang University. The study is compliant with all relevant ethical regulations regarding animal research. All experiments were conducted in a manner that minimized the number of animals used, and the suffering caused.

### CML patient samples collection

Patient samples were obtained from four newly diagnosed CML patients. Unstimulated bone marrow cells or blood were obtained upon initial diagnosis (prior to treatment). The diagnoses of CML were according to the 2016 WHO classification. This study was approved by the Ethics Committee of the First Affiliated Hospital, Zhejiang University School of Medicine. The study was conducted in compliance with the Helsinki Declaration. All patients provided informed consent for the use of samples for research purposes.

### Plasmids

Expression plasmids for ABL, BCR-ABL, and a series of mutants were kind gifts from the laboratories of Drs. Yong Cang, Shaoguang Li and Jean Y. Wang. ABL1 was amplified by PCR and subcloned into pRK5. Mammalian expression plasmids for HA- or FLAG-tagged Smads have been previously described.^8,11^ Smad4 Y195/301/322E and Smad4 Y195/301/322 F mutants were generated by PCR-based mutagenesis and confirmed by sequencing. Lentiviral expression plasmids for Smad4 and its mutants were obtained by subcloning into pXN-MBP.

### Antibodies and reagents

Antibodies used in this study were commercially obtained as the following: anti‐PAI-1 (C-9) antibody from Santa Cruz Biotech; anti‐p21 Waf1/Cip1 (2947 S), anti-p15 INK4B (4822 S), anti-p57/Kip2 (2557 S), anti-p-Smad2 (Ser465/467) /Smad3 (Ser423/425) (8828 S), anti-Smad2/3 (8685 S), anti-Smad4 (38454 S), anti‐HA (3724), anti-Myc (2276 S), anti-Caspase-3 (2662 S), anti-PARP (9532 S), anti-p-Tyrosine/P-Tyr-100 (9411 S), anti-ABL1 (2862 S), anti-Ac-Histone H3 (K9) (9649 S), anti-Ac-Histone H3 (K18) (9675 S) antibodies from Cell Signaling Technology; anti-KAT3B/p300 (3G230), anti-Histone H3 (ab1791) antibodies from Abcam; anti-GAPDH (G8795), anti-β-actin (A5441), anti-FLAG (F3165), rabbit IgG (I5006) and mouse IgG (I5381) from Sigma‐Aldrich. Polyclonal antibodies against Smad4 pY195, pY301, or pY322 were generated by GL Biochem (Shanghai) Ltd. TGF-β (TGFB1-100) was purchased from StemRD, SB431542 (S4317) from Sigma‐Aldrich and Gleevec/imatinib (CDS022173) from Sigma‐Aldrich, respectively.

### Cell culture and transfection

K562 cells were maintained in RPMI 1640 medium (Corning) supplemented with 10% fetal bovine serum (FBS) (Invitrogen). HaCaT cells were maintained in MEM-EBSS medium (Corning) with 10% FBS. HEK293T cells were cultured with DMEM medium (Corning) supplemented with 10% FBS. HaCaT cells were transiently transfected with X‐tremeGENE (Roche Applied Science) and HEK293T cells with PEI (Polyscience). K562 or HaCaT stable cell lines expressing ABL or Smad4 variants were generated by lentivirus infection and selected with puromycin/G418 at appropriate concentrations. Bone marrow samples were collected from CML patients. PBMCs were separated from bone marrow samples by Ficoll-Paque density gradient centrifugation (Miltenyi Biotec, Bergisch Gladbach, Germany). PBMCs were maintained in a-MEM medium (Corning) supplemented with 10% fetal bovine serum (FBS) (Invitrogen).

### Gene knockout and RNA interference

Generation of Smad4−/− HaCaT cells using CRISPR-Cas9 technology has been described in our previous study4. Smad4−/− K562 cells were similarly established. Smad4 knockout was verified by genotyping, RT-PCR, and anti-Smad4 western blotting.

To knockdown the expression of ABL1/BCR-ABL1, siRNAs targeting the ABL1 coding region were custom-made from Sigma and transfected into K562 cells using HiPerFect® Transfection Reagent (QIAGEN). The sequences of siRNAs were:

siABL1-1: GACCAACUUGUUCAGCGCC;

siABL1-2: GAAGGGAGGGUGUACCAUU.

### RNA extraction and quantitative RT-PCR (qRT-PCR) analysis

Total RNAs were extracted from cells using TRIzol reagent (Invitrogen), and 1 mg RNA was used for reverse transcription using the PrimeScript® RT reagent kit (TaKaRa). cDNA was diluted and used as a template for qRT-PCR analysis. Primers used in the qPCR are listed below (5′–3′):

h-Smad4-For: CCACCAAGTAATCGTGCATCG

h-Smad4-Rev: TGGTAGCATTAGACTCAGATGGG

h-PAI-1-For: CAAGAGTGATGGCAATGTGAC

h-PAI-1-Rev: TTTGCAGGATGGAACTACGG

h-p15-For: CAACGGAGTCAACCGTTTC

h-p15-Rev: TGAGAGTGGCAGGGTCTG

h-p21-For: ACCATGTGGACCTGTCACTGT

h-p21-Rev: TTAGGGCTTCCTCTTGGAGAA

h-p57-For: GCGGCGATCAAGAAGCTGT

h-p57-Rev: GCTTGGCGAAGAAATCGGAGA

h-ABL1-For: CCCAACCTTTTCGTTGCACTGT

h-ABL1-Rev: CGGCTCTCGGAGGAGACGTAGA

h-GAPDH-For: CGACCACTTTGTCAAGCTCA

h-GAPDH-Rev: TTACTCCTTGGAGGCCATGT

### ChIP assays

Cells were subject to formaldehyde crosslinking at room temperature for 10 min, after which the reaction was stopped by 2.5 M Glycine solution (final concentration: 0.2 M). Cells were collected by centrifugation at 3000 × *g* for 5 min and washed with cold PBS. Cell lysis, chromatin preparation, immunoprecipitation, and DNA quantitation were essentially conducted as described using ChIP assay kit from Thermo Scientific (No. 26156).

ChIP primers for specific genes are listed as follows (5′-3′):

h-PAI-1-For: GCAGGACATCCGGGAGAGA

h-PAI-1-Rev: CCAATAGCCTTGGCCTGAGA

h-p15-For: CTGCCTGGGGATGAATTTAAC

h-p15-Rev: GGTTTCACTGTGGAGACGTTG

h-p21-For: TGCTGGAACTCGGCCAGGCT

h-p21-Rev: AGCGCGGCCCTGATATACAAC

h-p57-For-1: CCTGCTGGAAGTCGTAATCC

h-p57-Rev-1: CACGATGGAGCGTCTTGT

h-p57-For-2: TCTCGCTGTCCTCTCCTCTC

h-p57-Rev-2: GCACTAGTACTGGGAAGGTC

### Immunoprecipitation and western blotting

Cells were harvested, washed with ice-cold PBS, and then lyzed in an ice-cold lysis buffer containing inhibitors of proteases and phosphatases. After centrifugation, the supernatants were incubated with protein A Sepharose (GE Healthcare) and a primary antibody at 4 °C for 4 h. Immunoprecipitated proteins was eluted in SDS sample loading buffer before gel electrophoresis and western blotting.

For western blotting, protein samples (either immunoprecipitated proteins or whole-cell lysates) were separated on SDS-PAGE and transferred to PVDF membranes (Millipore). The membranes were blocked in 5% non-fat milk, incubated with appropriate primary and HRP‐conjugated secondary antibodies, and then detected by chemiluminescence (Thermo Fisher).

### Immunofluorescence

Cells were cultured on coverslips in a 24-well plate with appropriate transfection and/or treatment as indicated in the figure legends or text. For immunofluorescence, cells were fixed in 4% paraformaldehyde for 20 min, permeablized with 0.5% Triton-X 100 for 15 min at room temperature, blocked in 5% BSA, incubated with primary antibodies, washed three times with PBS, and finally incubated with Alexa Fluor 546- or Alexa Fluor 488-conjugated secondary antibodies (Invitrogen). After 3× washing, coverslips were mounted with ProLong Gold antifade. Fluorescence images were observed and acquired by Zeiss LSM710 confocal microscope (Carl Zeiss).

### Reporter luciferase assay

Smad3/Smad4-responsive reporters SBE-Luc and CAGA-Luc have been previously described.^24^ Cells were transfected with expression plasmids for ABLs and luciferase reporters as indicated in the figure legends. 24 h after transfection, cells were treated with TGF-b1 (2 ng/mL, 8 h), harvested, and lyzed in Passive lysis buffer (Promega). The expression of luciferase reporters was analyzed with the Dual-Luciferase Reporter Assay System (Promega, USA). All assays were done and repeated in triplicates, and all values were normalized for transfection efficiency against Renilla luciferase activities.

### Statistical analysis

All the statistical results were analyzed with three replicates according to a completely randomized design. All data were performed by Analysis of variance (ANOVA). Data were analyzed statistically by repeated measures using SPSS 17.0 procedures, and *p* < 0.05 was considered statistically significant.

## Supplementary information

Supplementary figures

## Data Availability

All data generated or analyzed during this study are included in this article or in the supplementary information files. All materials in this article are available upon reasonable request from the corresponding authors.
