# Supplementary material for: Imatinib blocks tyrosine phosphorylation of Smad4 and restores TGF-β growth-suppressive signaling in BCR-ABL1-positive leukemia
Source: Signal Transduct Target Ther. 2023 Mar 24;8:120. doi: 10.1038/s41392-023-01327-5 (PMC10036327; doi:10.1038/s41392-023-01327-5)
Supplement: Supplementary file 1 — Supplementary figures [file 41392_2023_1327_MOESM1_ESM.docx]

Supplementary Materials for

**Imatinib blocks tyrosine phosphorylation of Smad4 and restores TGF-β growth-suppressive signaling in BCR-ABL1-positive leukemia**

Lijing Wang^1,2,3,*^, Shuchen Gu^1,2,3,*^, Fenfang Chen^1,*^, Yi Yu^1,2,3^, Jin Cao^1,2,3^, Xinran Li^1,2,3^, Chun Gao^1,2,4^, Yanzhen Chen^1^, Shuchong Yuan^4^, Xia Liu^5^, Jun Qin^6^, Bin Zhao^1,2,3^, Pinglong Xu^1,2,3^, Tingbo Liang^7^, Hongyan Tong^4^, Xia Lin^7,#^, and Xin-Hua Feng^1,2,3,8,#^

^1^ The MOE Key Laboratory of Biosystems Homeostasis & Protection and Zhejiang Provincial Key Laboratory of Cancer Molecular Cell Biology, Life Sciences Institute, Zhejiang University, Hangzhou, Zhejiang 310058, China.

^2^ Center for Life Sciences, Shaoxing Institute, Zhejiang University, Shaoxing, Zhejiang 321000, China.

^3^ Cancer Center, Zhejiang University, Hangzhou, Zhejiang 310058, China.

^4^ Department of Hematology, The First Affiliated Hospital, Zhejiang University School of Medicine, Hangzhou 310003, China.

^5^ ZJU-Hangzhou Global Scientific and Technological Innovation Center, Zhejiang University, Hangzhou, Zhejiang 311200, China.

^6^ Beijing Proteome Research Center, National Center for Protein Sciences, Beijing, China.

^7^ Department of Hepatobiliary and Pancreatic Surgery and Zhejiang Provincial Key Laboratory of Pancreatic Disease, The First Affiliated Hospital, Zhejiang University School of Medicine, Hangzhou 310003, China.

^8^ The Second Affiliated Hospital, Zhejiang University, Hangzhou, Zhejiang 310009, China.

^*^ These authors contributed equally.

^#^ To whom correspondence may be addressed. E-mail: [xialinzheda@zju.edu.cn](mailto:xialinzheda@zju.edu.cn); [fenglab@zju.edu.cn](mailto:fenglab@zju.edu.cn)

**This PDF file includes:**

Figures. S1 to S7


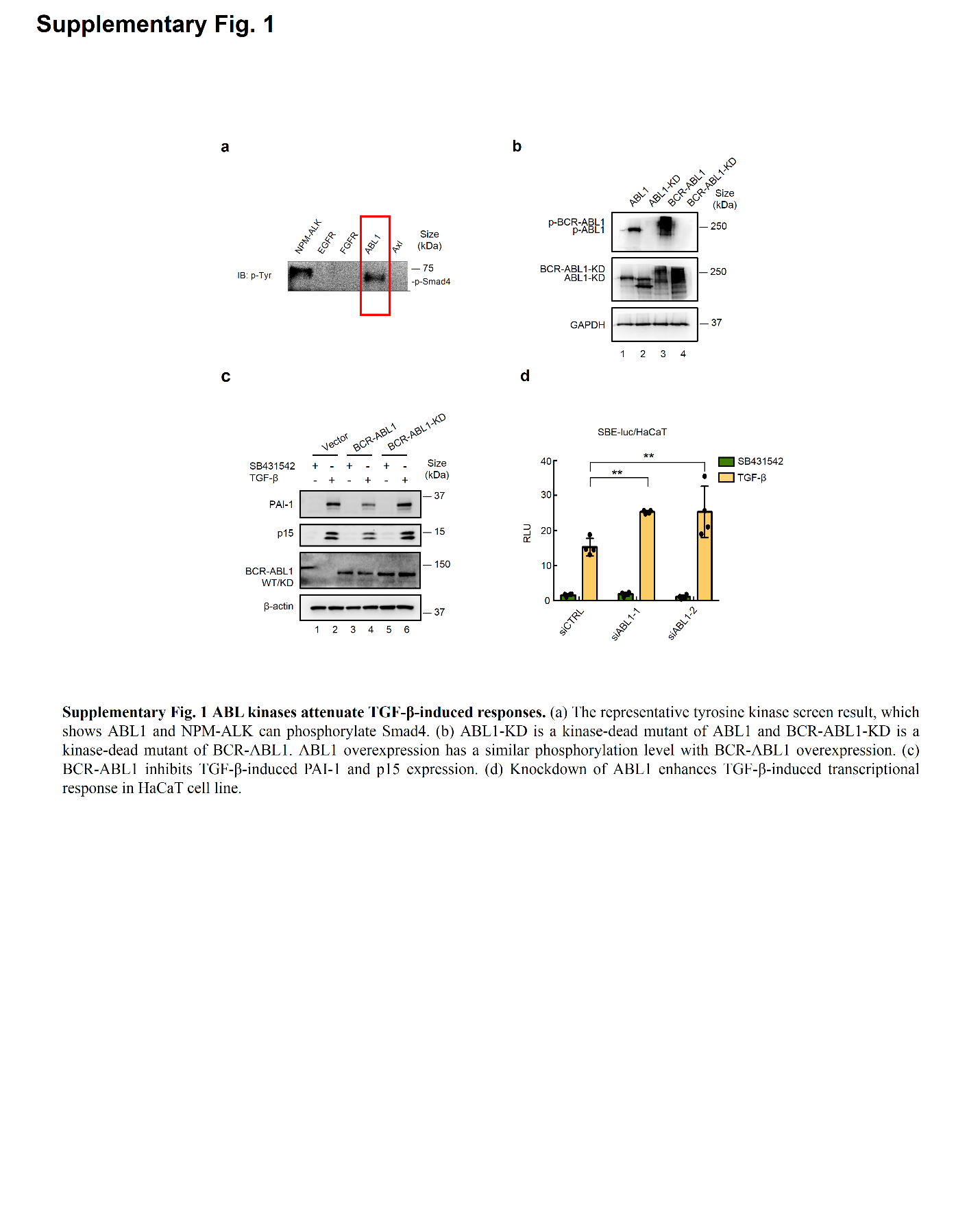


Figure. S1. ABL kinases attenuate TGF-β-induced responses. (a) The representative tyrosine kinase screen shows that ABL1 and NPM-ALK can phosphorylate Smad4. (b) Overexpression ABL1 or BCR-ABL1 can auto-phosphorylate. ABL1-KD and BCR-ABL1-KD are kinase-dead mutants of ABL1 and BCR-ABL1, respectively. (c) BCR-ABL1, but not BCR-ABL1-KD, inhibits TGF-β-induced PAI-1 and p15 expression. (d) Knockdown of ABL1 enhances TGF-β-induced SBE reporter response in HaCaT cells.


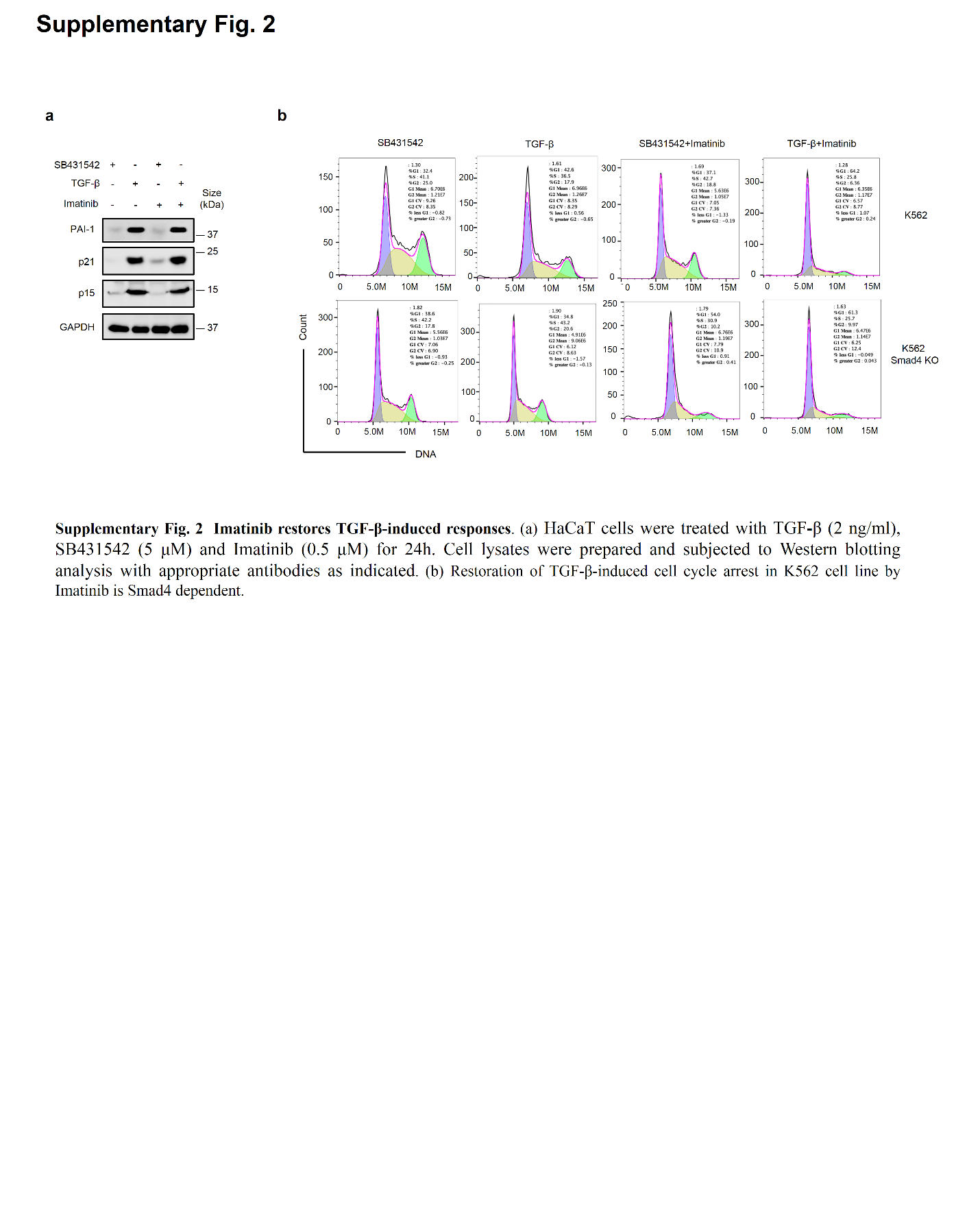


Figure. S2. Imatinib restores TGF-β-induced responses. (a) Imatinib has no effects on TGF-induced responses in BCR-ABL1-negative HaCaT cells. Cells were treated with TGF-β (2 ng/ml), SB431542 (5 μM) and Imatinib (0.5 μM) for 24h. Cell lysates were prepared and subjected to Western blotting analysis with appropriate antibodies as indicated. (b) Imatinib restores TGF-β-induced Smad4-dependent cell cycle arrest in K562 cells.


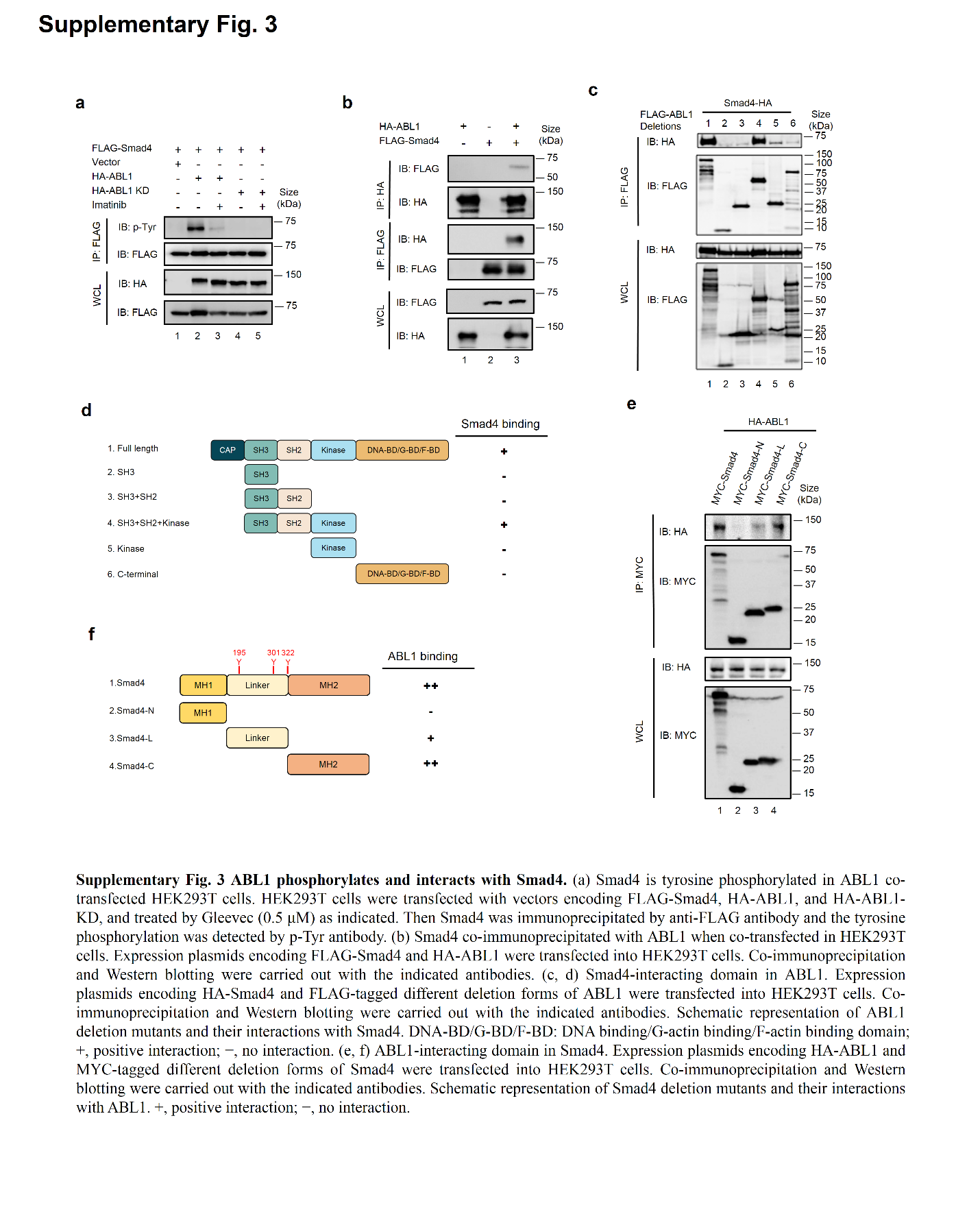


Figure. S3. ABL1 phosphorylates and interacts with Smad4. (a) Smad4 is tyrosine phosphorylated in ABL1 co-transfected HEK293T cells. HEK293T cells were transfected with vectors encoding FLAG-Smad4, HA-ABL1, and HA-ABL1-KD, and treated by Imatinib (0.5 μM) as indicated. Smad4 protein was then immunoprecipitated by an anti-FLAG antibody and the tyrosine phosphorylation was detected by Western blotting using a p-Tyr antibody. (b) Smad4 interacts with ABL1. Expression plasmids encoding FLAG-Smad4 and HA-ABL1 were transfected into HEK293T cells. Co-immunoprecipitation and Western blotting were carried out with the indicated antibodies. (c) Mapping of Smad4-interacting domains in ABL1. Expression plasmids encoding HA-Smad4 and FLAG-tagged different deletion forms of ABL1 were transfected into HEK293T cells. Co-immunoprecipitation and Western blotting were carried out with the indicated antibodies. (d) Schematic representation of ABL1 deletion mutants and their interactions with Smad4. DNA-BD/G-BD/F-BD: DNA binding/G-actin binding/F-actin binding domain; +, positive interaction; −, no interaction. (e) Mapping of ABL1-interacting domains in Smad4. Expression plasmids encoding HA-ABL1 and MYC-tagged different deletion forms of Smad4 were transfected into HEK293T cells. Co-immunoprecipitation and Western blotting were carried out with the indicated antibodies. (f) Schematic representation of Smad4 deletion mutants and their interactions with ABL1. +, positive interaction; −, no interaction.


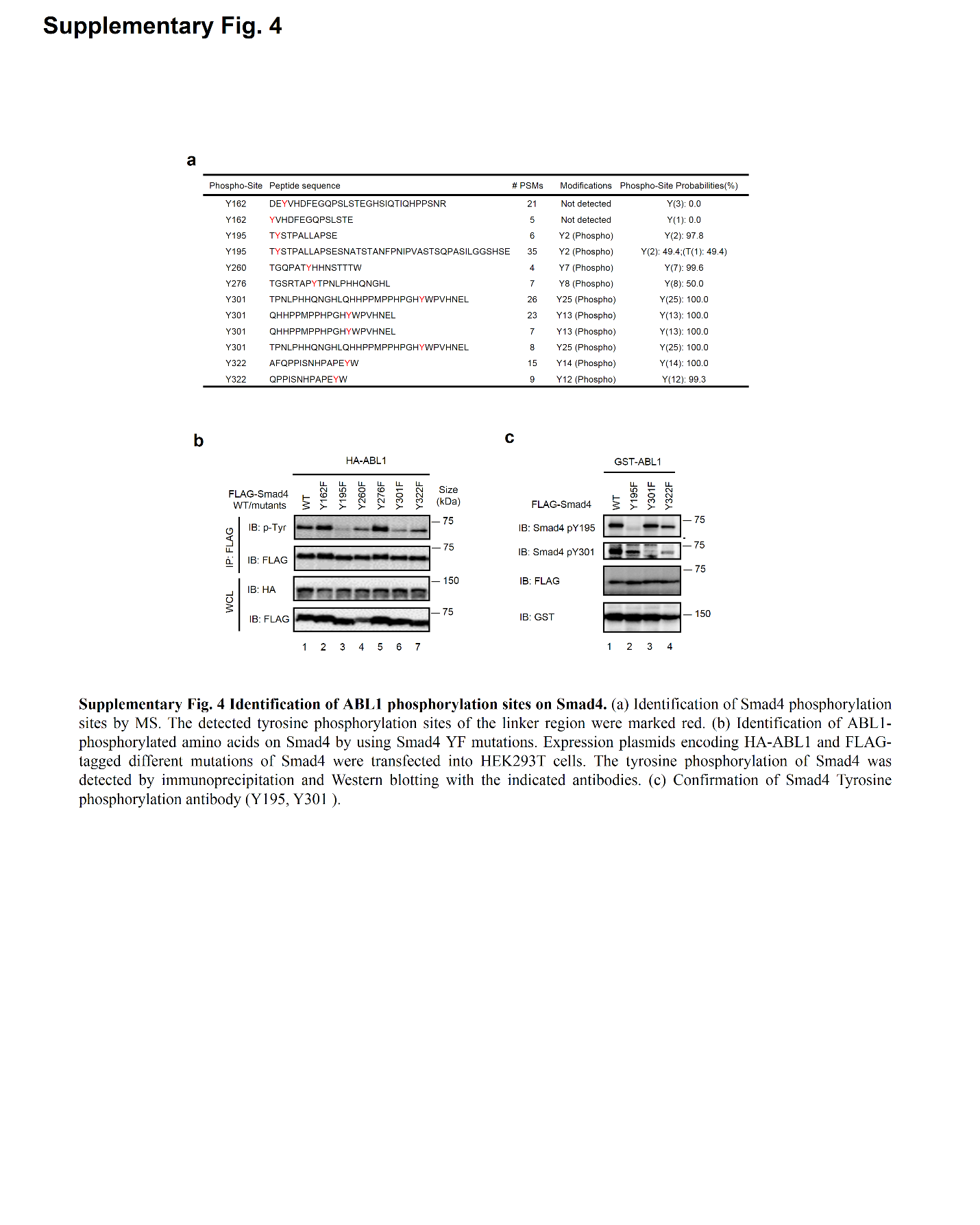


Figure. S4. 4 Identification of ABL1 phosphorylation sites on Smad4. (a) Identification of Smad4 phosphorylation sites by mass spectrometry. The detected tyrosine phosphorylation sites of the linker region were marked with red Y. (b) Identification of ABL1-phosphorylated amino acids on Smad4 by using Smad4 YF mutations. Expression plasmids encoding HA-ABL1 and FLAG-tagged Smad4 mutants were transfected into HEK293T cells. The tyrosine phosphorylation of Smad4 was detected by immunoprecipitation and Western blotting with the indicated antibodies. (c) Recombinant GST-ABL1 can phosphorylate Smad4 tyr-195 and -301. Smad4pY195 and Smad4pY301 are site-specific antibodies.


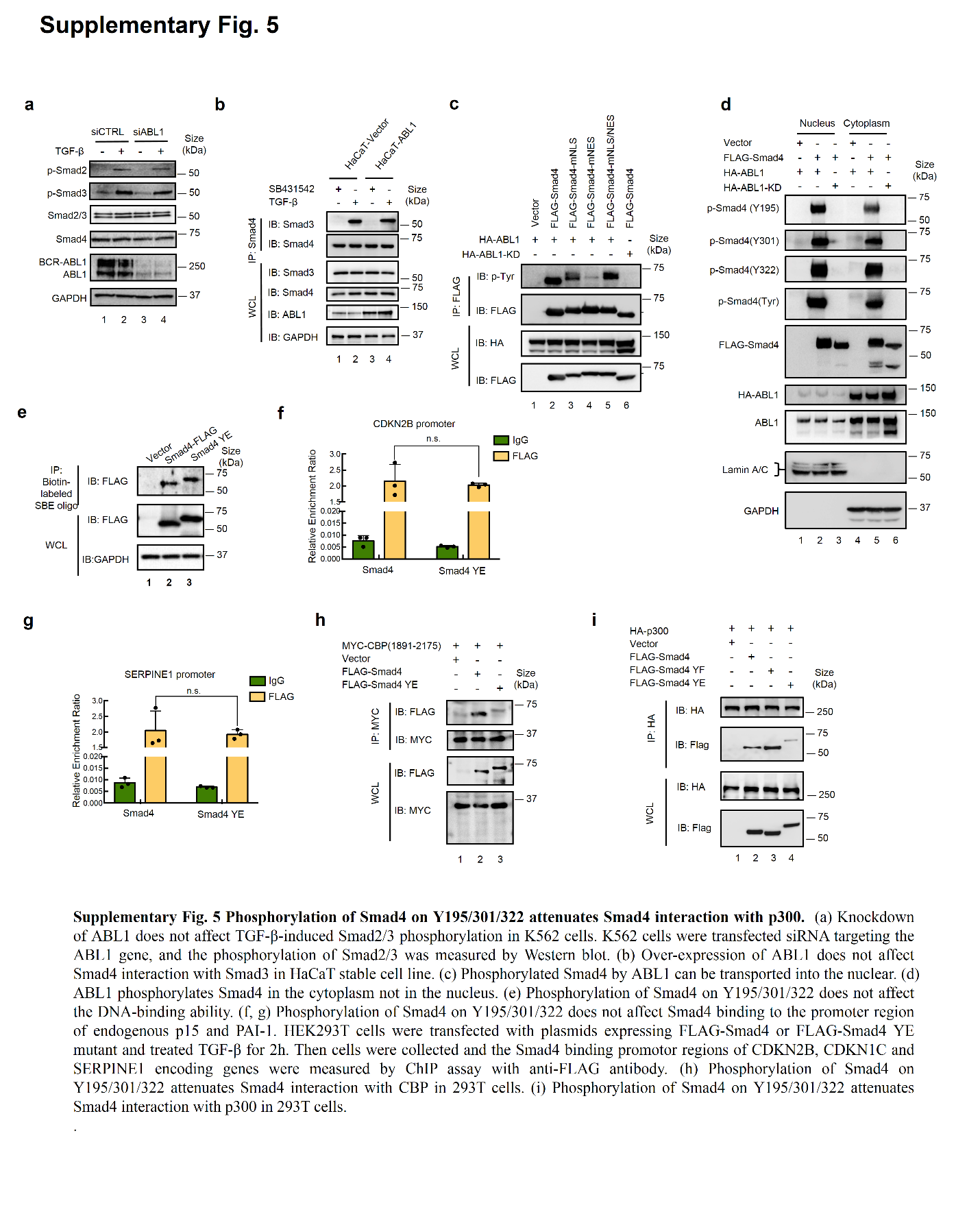


Figure. S5. Phosphorylation of Smad4 on Y195/301/322 attenuates the interaction of Smad4 with transcription coactivator p300/CBP. (a) ABL1 does not affect TGF-β-induced Smad2/3 phosphorylation in K562 cells. K562 cells were transfected with an siRNA targeting ABL1, and subjected to measurement of Smad2/3 phosphorylation by Western blotting. (b) ABL1 does not affect Smad4 interaction with Smad3 in HaCaT cells. ABL1 was stably expressed in HaCaT-ABL1 cells. (c) ABL1 Phosphorylates Smad4 in the cytoplasm. mNLS and mNES are Smad4 mutants harboring mutation in the NLS and NES, respectively. (d) ABL1-phosphorylated Smad4 can undergo nucleocytoplasmic shuttling. (e) Phospho-mimicking Smad4 YE (Y195E/Y301E/Y322E) retains its DNA-binding ability in a DNA pulldown assay. (f, g) Smad4 and Smad4 YE can similarly bind the promoters of CDKN2B and SERPINE1. HEK293T cells were transfected with plasmids expressing FLAG-Smad4 or FLAG-Smad4 YE mutant and treated TGF-β for 2h. The binding of Smad4 to the promotors was measured by ChIP assay using an anti-FLAG antibody. (h) Smad4 YE has reduced interaction with CBP. HEK293T cells were transfected with plasmids expressing FLAG-Smad4 or FLAG-Smad4 YE mutant and MYC-CBP (1891-2175). The Smad4-CBP interaction was analyzed by IP-Western blotting. (i) Smad4 YE has reduced interaction with p300. Cell transfection and IP-Western blotting analysis were done as in panel h.


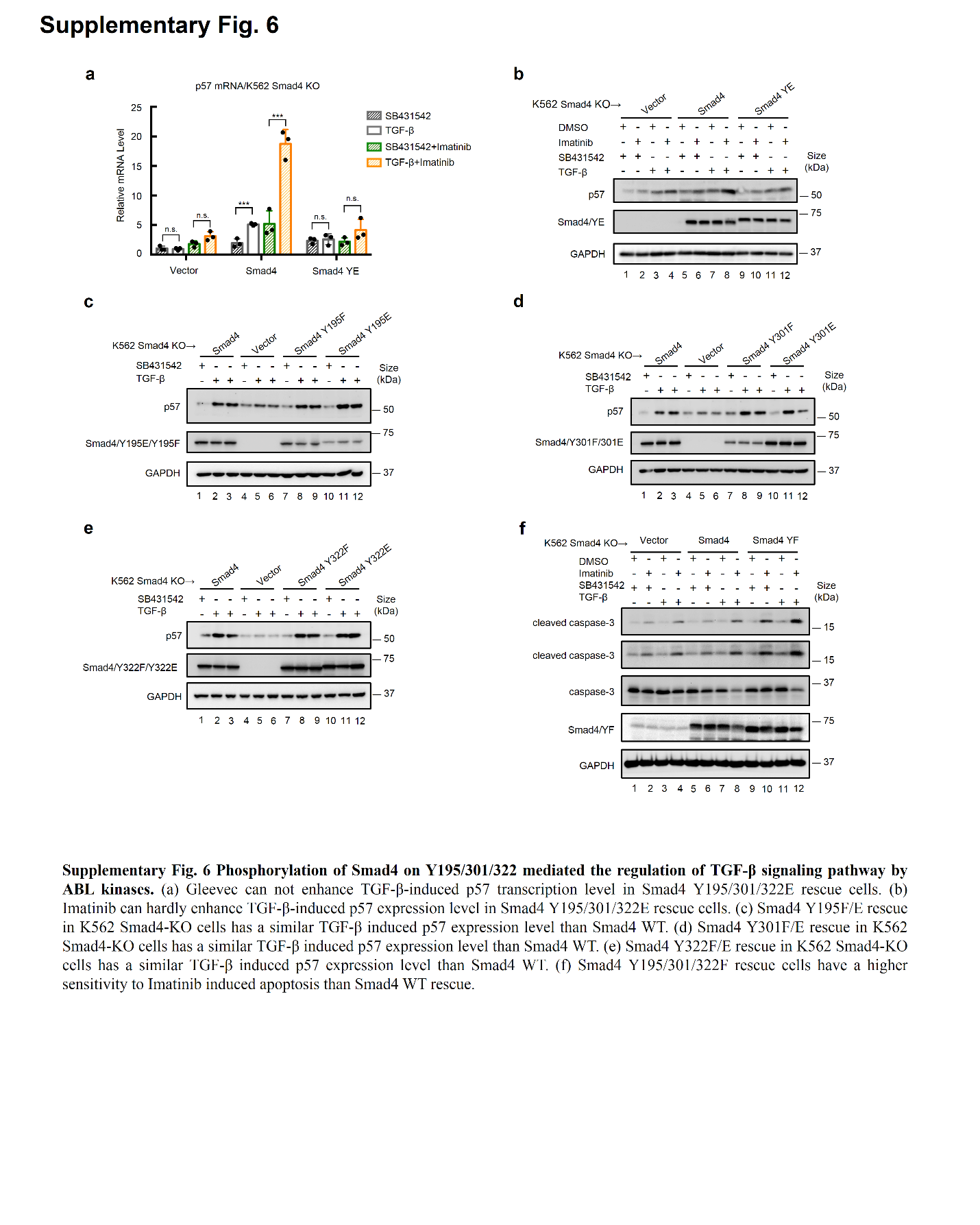


Figure. S6. Analysis of Smad4 Y195/301/322 single or triple phosphorylation on TGF-β responses. (a) Imatinib cannot enhance TGF-β-induced p57 transcription level in Smad4 YE cells. (b) Imatinib weakly enhances TGF-β-induced p57 level in Smad4 YE cells. (c) Smad4 WT, Smad4 Y195F or Y195E similarly rescues TGF-β-induced p57 expression level in K562 Smad4-KO cells. (d) Smad4 WT, Smad4 Y301F or Y301E similarly rescues TGF-β-induced p57 expression level in K562 Smad4-KO cells. (e) Smad4 WT, Smad4 Y322F or Y322E similarly rescues TGF-β-induced p57 expression level in K562 Smad4-KO cells. (f) Imatinib induces a higher level of caspase-3 cleavage in K562 Smad4-KO cells re-expressing Smad4 triple YF mutant (Y195F/Y301F/Y322F) than Smad4 WT.


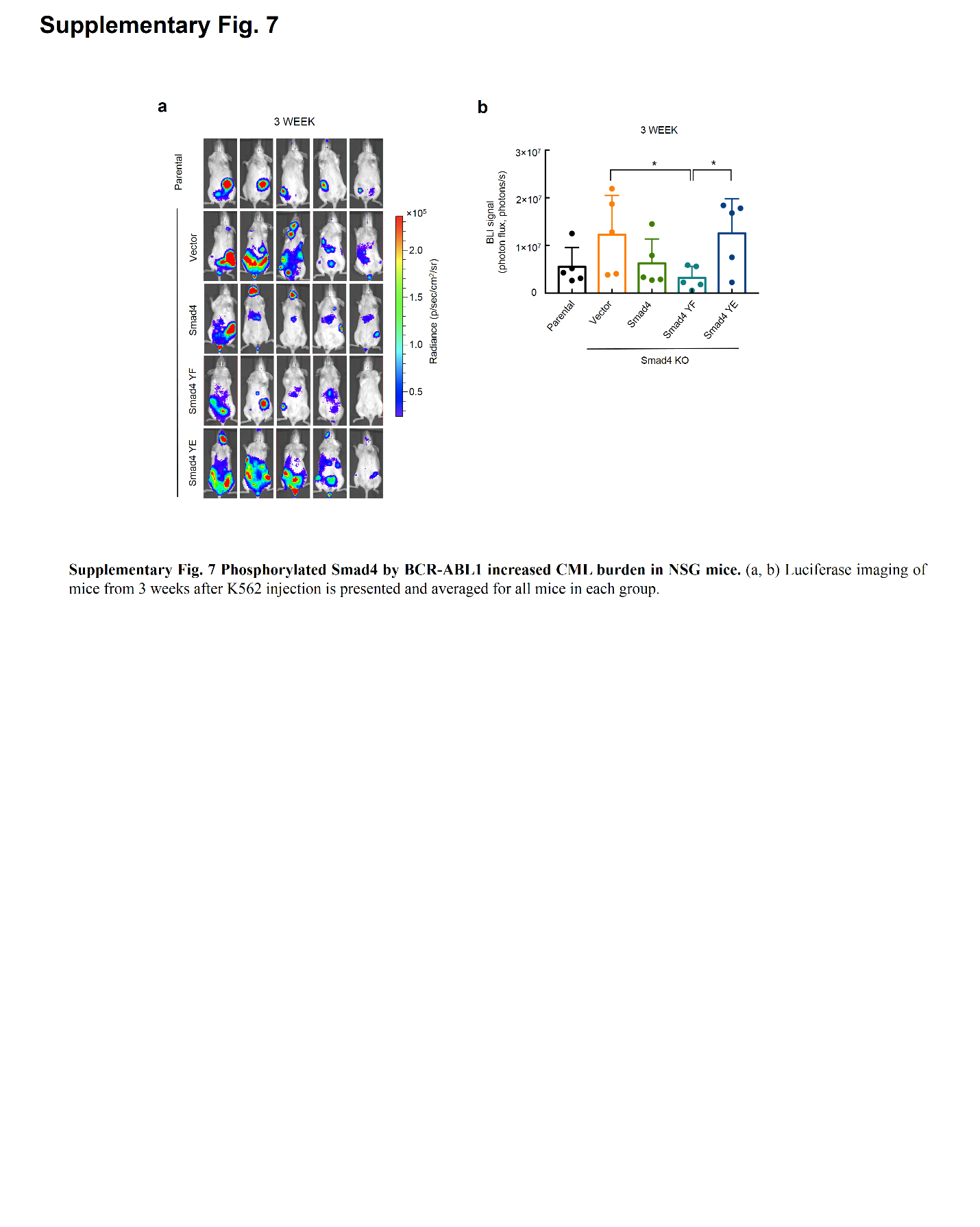


Figure. S7. Smad4 YE increases CML burden in NSG mice. (a) Luciferase imaging of mice upon K562 injection for 3 weeks. (b) Statistical analysis of BLI quantitation in panel a.
